# Fecal Microbiome Reflects Disease State and Prognosis in Inflammatory Bowel Disease in an Adult Population-Based Inception Cohort

**DOI:** 10.1093/ibd/izaf060

**Published:** 2025-04-25

**Authors:** Simen Hyll Hansen, Maria Gjerstad Maseng, Olle Grännö, Marie V Vestergaard, Corinna Bang, Bjørn C Olsen, Charlotte Lund, Christine Olbjørn, Emma E Løvlund, Florin B Vikskjold, Gert Huppertz-Hauss, Gøri Perminow, Hussain Yassin, Jørgen Valeur, Kristina I Aass Holten, Magne Henriksen, May-Bente Bengtson, Petr Ricanek, Randi Opheim, Raziye Boyar, Roald Torp, Svein O Frigstad, Tone Bergene Aabrekk, Trond Espen Detlie, Vendel A Kristensen, Vibeke Strande, Øistein Hovde, Øyvind Asak, Tine Jess, Andre Franke, Jonas Halfvarsson, Marte L Høivik, Johannes R Hov

**Affiliations:** Norwegian PSC Research Center, Department of Transplantation Medicine, Division of Surgery, Inflammatory Diseases and Transplantation, Oslo University Hospital, Oslo, Norway; Institute of Clinical Medicine, Faculty of Medicine, University of Oslo, Oslo, Norway; Research Institute of Internal Medicine, Division of Surgery, Inflammatory Diseases and Transplantation, Oslo University Hospital, Oslo, Norway; Institute of Clinical Medicine, Faculty of Medicine, University of Oslo, Oslo, Norway; Department of Gastroenterology, Oslo University Hospital, Oslo, Norway; Bio-Me, Oslo, Norway; School of Medical Sciences, Örebro University, Örebro, Sweden; Center for Molecular Prediction of Inflammatory Bowel Disease, PREDICT, Department of Clinical Medicine, Aalborg University, Copenhagen, Denmark; Institute of Clinical Molecular Biology, Christian-Albrechts-University of Kiel, Kiel, Germany; Institute of Clinical Medicine, Faculty of Medicine, University of Oslo, Oslo, Norway; Department of Gastroenterology, Telemark Hospital, Skien, Norway; Institute of Clinical Medicine, Faculty of Medicine, University of Oslo, Oslo, Norway; Department of Gastroenterology, Oslo University Hospital, Oslo, Norway; Department of Pediatric and Adolescent Medicine, Akershus University Hospital, Lørenskog, Norway; Department of Pediatric and Adolescent Medicine, Østfold Hospital Trust, Kalnes, Norway; Department of Pediatric and Adolescent Medicine, Drammen Hospital, Vestre Viken Hospital Trust, Drammen, Norway; Department of Gastroenterology, Telemark Hospital, Skien, Norway; Department of Pediatrics, Oslo University Hospital, Oslo, Norway; Department of Pediatrics, Telemark Hospital Kjørbekk, Skien, Norway; Institute of Clinical Medicine, Faculty of Medicine, University of Oslo, Oslo, Norway; Unger-Vetlesen Institute, Lovisenberg Diaconal Hospital, Oslo, Norway; Institute of Clinical Medicine, Faculty of Medicine, University of Oslo, Oslo, Norway; Department of Gastroenterology, Østfold Hospital Trust, Grålum, Norway; Department of Gastroenterology, Østfold Hospital Trust, Grålum, Norway; Department of Gastroenterology, Vestfold Hospital Trust, Tonsberg, Norway; Department of Gastroenterology, Lovisenberg Diaconal Hospital, Oslo, Norway; Department of Gastroenterology, Oslo University Hospital, Oslo, Norway; Department of Nursing Science, Institute of Health and Society, University of Oslo, Oslo, Norway; Department of Medicine, Diakonhjemmet Hospital, Oslo, Norway; Medical Department, Innlandet Hospital Trust, Hamar, Norway; Department of Medicine, Bærum Hospital, Vestre Viken Hospital Trust, Gjettum, Norway; Institute of Clinical Medicine, Faculty of Medicine, University of Oslo, Oslo, Norway; Department of Gastroenterology, Vestfold Hospital Trust, Tonsberg, Norway; Institute of Clinical Medicine, Faculty of Medicine, University of Oslo, Oslo, Norway; Department of Gastroenterology, Akershus University Hospital, Lørenskog, Norway; Institute of Clinical Medicine, Faculty of Medicine, University of Oslo, Oslo, Norway; Department of Gastroenterology, Oslo University Hospital, Oslo, Norway; Department of Gastroenterology, Lovisenberg Diaconal Hospital, Oslo, Norway; Institute of Clinical Medicine, Faculty of Medicine, University of Oslo, Oslo, Norway; Department of Medicine, Innlandet Hospital Trust, Gjøvik, Norway; Department of Gastroenterology, Innlandet Hospital Trust, Lillehammer, Norway; Center for Molecular Prediction of Inflammatory Bowel Disease, PREDICT, Department of Clinical Medicine, Aalborg University, Copenhagen, Denmark; Department of Gastroenterology & Hepatology, Aalborg University Hospital, Aalborg, Denmark; Institute of Clinical Molecular Biology, Christian-Albrechts-University of Kiel, Kiel, Germany; Department of Gastroenterology, Faculty of Medicine and Health, Örebro University, Örebro, Sweden; Institute of Clinical Medicine, Faculty of Medicine, University of Oslo, Oslo, Norway; Department of Gastroenterology, Oslo University Hospital, Oslo, Norway; Norwegian PSC Research Center, Department of Transplantation Medicine, Division of Surgery, Inflammatory Diseases and Transplantation, Oslo University Hospital, Oslo, Norway; Institute of Clinical Medicine, Faculty of Medicine, University of Oslo, Oslo, Norway; Research Institute of Internal Medicine, Division of Surgery, Inflammatory Diseases and Transplantation, Oslo University Hospital, Oslo, Norway; Section of Gastroenterology, Department of Transplantation Medicine, Division of Surgery, Inflammatory Diseases and Transplantation, Oslo University Hospital, Oslo, Norway

**Keywords:** machine learning, ulcerative colitis, Crohn’s disease, IBSEN

## Abstract

**Introduction:**

We aimed to determine the diagnostic and prognostic potential of baseline microbiome profiling in inflammatory bowel disease (IBD).

**Methods:**

Participants with ulcerative colitis (UC), Crohn’s disease (CD), suspected IBD, and non-IBD symptomatic controls were included in the prospective population-based cohort Inflammatory Bowel Disease in South-Eastern Norway III (third iteration) based on suspicion of IBD. The participants donated fecal samples that were analyzed with 16S rRNA sequencing. Disease course severity was evaluated at the 1-year follow-up. A stringent statistical consensus approach for differential abundance analysis with 3 different tools was applied, together with machine learning modeling.

**Results:**

A total of 1404 individuals were included, where *n* = 1229 samples from adults were used in the main analyses (*n* = 658 UC, *n* = 324 CD, *n* = 36 IBD-U, *n* = 67 suspected IBD, and *n* = 144 non-IBD symptomatic controls). Microbiome profiles were compared with biochemical markers in machine learning models to differentiate IBD from non-IBD symptomatic controls (area under the receiver operating curve [AUC] 0.75-0.79). For UC vs controls, integrating microbiome data with biochemical markers like fecal calprotectin mildly improved classification (AUC 0.83 to 0.86, *P < *.0001). Extensive differences in microbiome composition between UC and CD were identified, which could be quantified as an index of differentially abundant genera. This index was validated across published datasets from 3 continents. The UC-CD index discriminated between ileal and colonic CD (linear regression, *P = .*008) and between colonic CD and UC (*P = .*005), suggesting a location-dependent gradient. Microbiome profiles outperformed biochemical markers in predicting a severe disease course in UC (AUC 0.72 vs 0.65, *P < *.0001), even in those with a mild disease at baseline (AUC 0.66 vs 0.59, *P *< .0001).

**Conclusions:**

Fecal microbiome profiling at baseline held limited potential to diagnose IBD from non-IBD compared with standard-of-care. However, microbiome shows promise for predicting future disease courses in UC.

**Key Messages**

- What is already known?

Inflammatory bowel disease (IBD) typically presents with an altered fecal microbiome, but the exact alterations often differ in the literature due to low sample sizes and lenient methods of analysis, masking the real diagnostic and prognostic value.

- What is new here?

We show that microbial alterations relate to diagnosis and prognosis in a population-based inception cohort of >1500 newly diagnosed cases of IBD. In particular, microbiome profiles separate ulcerative colitis (UC) from Crohn’s disease and predict severe outcomes in UC.

- How can this study help patient care?

Microbiome profiling has the potential to aid diagnostics and prognostics by providing information beyond currently available markers of inflammation.

## Introduction

The gut microbiome likely plays a role in the pathogenesis of inflammatory bowel disease (IBD).^1^ Experimental studies show that colitis in mice can be transferred via gut microbiome.^2^ There are observations of a diagnostically predictive value of microbiome profiles (the collection of relative bacterial abundances per sample) in pediatric Crohn’s disease (CD),^3^ and there is some evidence that fecal microbiota transplantation may induce remission of ulcerative colitis (UC).^4^ Despite the large potential and many years of research, the gut microbiome so far does not have an important role in solving clinical challenges in modern IBD care.

Inflammatory bowel disease has had a rising incidence and prevalence worldwide,^5^ suggesting an influence of environmental factors that could in part be microbial. The clinical challenges related to IBD include diagnostic delays, difficulties in disease phenotyping, and ineffective risk stratification.^6,7^ Current noninvasive biomarkers used in IBD diagnosis and follow-up are unspecific^8^ and a diagnosis of IBD necessitates invasive procedures, which are expensive, low-throughput, and uncomfortable. There is a need for early identification of people with poor prognosis to initiate effective treatment and avoid long-term complications.^6,9^ Simultaneously, early identification must be specific enough to avoid over-treatment. As new and expensive targeted therapies are introduced continuously, these aspects will become even more important.

Compared to the healthy population, people with IBD typically present with a less diverse and more unstable microbiome,^10^ with a distinct depletion of bacterial species associated with gut health^11–13^ together with an increase in opportunistic pathogens.^13–16^ However, inconclusive or divergent findings are common in microbiome research,^15^ which has limited translational efforts to clinical utility. A recent systematic review of IBD microbiome studies^15^ uncovered several important limitations in the literature that may explain its heterogeneity. The authors found that 58% of studies were underpowered (<30 participants with IBD), 83% of studies had a biased selection of controls, and 66% of studies did not adjust for multiple comparisons, emphasizing the urgency of conducting large-scale studies with robust and reproducible methods.

The aim of the present study was to confirm the hypothesis that newly diagnosed people with IBD have an altered gut microbiome, and that measuring the fecal microbiome holds diagnostic and prognostic value when analyzed with robust and reproducible methods. We conducted a large-scale microbiome analysis at baseline in the large Norwegian prospective inception cohort IBSEN III (Inflammatory Bowel Disease in South-Eastern Norway),^17^ proposing that the use of a population-based design, clinically relevant controls and employing stringent differential abundance (DA) methods including ANCOM-BC2, and state-of-the-art analytical approaches within machine learning, may demonstrate the true translational value of the fecal microbiome. In the current study, we refer to the collection of identified bacterial taxa as the microbiome. Our findings suggest that the fecal microbiome is closely related to the current state and location of intestinal inflammation and may predict future severe disease course in UC, while it is not competitive as a diagnostic tool to separate people with IBD from symptomatic people without IBD.

## Materials and Methods

### Study Population, Clinical Definitions, and Biochemical Markers

All participants of the IBSEN III study who donated fecal samples at inclusion were included in the present study. Inflammatory Bowel Disease in South-Eastern Norway III is a population-based observational inception cohort, which recruited people who had been referred to investigation for suspected IBD during the years 2017-2019 at all hospitals in the health region of South-Eastern Norway. Recruitment and data collection in IBSEN III are described in detail by Kristensen et al.^17^ After diagnostic work-up, which included stool samples for analysis of pathogens, blood samples, endoscopy with biopsies, and if indicated magnetic resonance imaging (MRI) or computed tomography (CT), UC and CD diagnosis was determined according to internationally accepted criteria.^18^ Those with apparent, mild inflammation at endoscopy or MRI/CT, but who did not meet the diagnostic criteria for IBD were defined as suspected IBD. People who had normal diagnostic work-up including a normal endoscopy were defined as non-IBD symptomatic controls.

Clinical, biochemical, endoscopic, and demographic data were collected at baseline and at the 1-year follow-up according to protocol.^17,19^ We used the diagnosis given at baseline for our analysis; however, a small proportion received a revised classification at follow-up due to endoscopy and radiology, and these cases were investigated specifically. Clinical disease activity was measured using the Simple Clinical Colitis Activity Index (SCCAI)^20^ for UC and the Harvey-Bradshaw Index (HBI)^21^ for CD, and disease phenotype was defined according to the Montreal classification.^22^ Endoscopic scoring employed the Mayo endoscopic subscore for UC^23^ and a modified version of the Simple Endoscopic Score for CD.^24^ All of these are together referred to as clinical features.

Biochemical markers were obtained and recorded, including C-reactive protein (CRP), serum transferrin, serum ferritin, blood hemoglobin, serum albumin, and fecal calprotectin (F-calprotectin). Routine blood sample analyses were conducted locally, while F-calprotectin analysis was performed at the same laboratory for all participants, using Bühlmann Calprotectin ELISA EK-CAL, with results recorded within the range of 29-1801 µg/g. Results outside this range were recorded at the range’s lower or upper limit.

### Definition of Severe Disease Course

We applied a composite endpoint for severe disease course, referred to as “aggressive course” in prior research,^25^ reflecting difficult-to-control inflammation or complications. In short, severe disease course in UC was defined by either IBD-related hospitalization, surgery, stoma formation, more than 2 corticosteroid courses, or the use of 2 biologics within the first 12 months due to failure of the first. In CD, the criteria were similar, with additional inclusion of new findings or surgeries for fistulas, abscesses, or strictures.

### Fecal Sample Collection

The participants were asked to provide fecal samples at the time of inclusion, either before or at least 3 days after endoscopy. The fecal collection kit included one stool collection tube with DNA stabilizer (Invitek Diagnostics) for microbiome analysis and one dry tube for F-calprotectin analysis. A subset also received a Bristol Stool Scale (BSS) form. The stool collection was performed at home by the participants, and the samples were returned to the hospital by mail in ambient temperature. Upon receiving samples, they were stored at −80 °C until further processing. Healthy control fecal samples were available from a subset (*n* = 72) of a previously described cohort^26^ for processing and sequencing alongside the IBSEN III samples.

### Fecal Sample Processing and Sequencing

A detailed description of fecal sample processing is provided in the Supplementary Methods. In summary, DNA from homogenized samples was isolated in 2 batches on the QIAcube system before the V3-V4 region of the 16S rRNA gene was amplified and prepared for sequencing according to standard protocol.^27^ Inflammatory Bowel Disease in South-Eastern Norway III samples were not organized by disease groups, thereby hindering diagnosis-associated batch effects. After normalization and quality control, the amplicons were pooled into 8 libraries, before sequencing with the Illumina MiSeq platform and v3 kit (Illumina) at the Norwegian Sequencing Centre in Oslo. Samples yielding less than 10 000 reads were re-sequenced.

### Bioinformatics Processing

The full bioinformatics pipeline is described in detail in Supplementary Methods. Briefly, the sequences were filtered, demultiplexed, trimmed, and merged using bbduk, cutadapt, and bbmerge.^28–30^ Next, they were denoised using Deblur as implemented in *QIIME2 2021.4*^31,32^ and classified with the SILVA 138 database.^33–35^ Contaminants were removed before the remaining sequences were collapsed to the genus level and exported from *QIIME2* for subsequent analysis and modeling. All further filtering, rarefying, and analysis were done in R *4.2.3*^36^ and visualized with the R package *ggplot2 3.4.1*,^37^ unless stated otherwise.

### Validation Cohorts

A collection of IBD datasets processed and described by Vestergaard et al.^38^ was included to validate observed differences between CD and UC. Only datasets that were freely available were used ( Table S1). Additionally, validation statistics were calculated per continent, and thus we included only datasets from continents with sufficient data from both people with UC and CD. In total, 1998 IBD samples were used for validation, originating from North America (*n* = 800), Europe (*n* = 805), and Asia (*n* = 393). Description of IBD disease location was not available in these datasets.

### Bioinformatic Analysis and Statistics

Detailed confounder assessment and technical details of the analysis are described in the Supplementary Methods. We examined several potential factors that could affect our results and ultimately accounted for age, sex, antibiotics use (within 3 months prior to study inclusion), sampling delay (time from diagnosis to fecal sample received at biobank), and library (sequencing batch). The healthy controls were sequenced in a single library, and to account for this, the direct comparisons involving the healthy controls were limited to IBSEN III samples sequenced within the same library. In other analyses, the sequencing library was included as a random effect in mixed-effect models. Bristol stool scale evaluations existed for a subset of samples and were only included in a subset of analyses. Intra-individual (alpha) diversity indices (Shannon diversity index and the total number of observed taxa per sample) were calculated on a version of the dataset rarefied (subsampled) to 9503 reads. Alpha diversity was compared between groups using generalized linear models. Comparative compositionality of samples (beta diversity) was examined using Bray-Curtis dissimilarities on rarefied data and tested using a permutational multivariate analysis of variance (PERMANOVA) model, as well as permutational Analysis of multivariate dispersions (PERMDISP) to assess differences in dispersion between groups.

For comparisons of the relative abundance of different taxa (DA testing), we used a consensus-based approach as recommended by Nearing et al.^39^ Three methods were chosen based on their ability to account for sequencing depth heterogeneity: *ANCOM-BC2 2.0.3*,^40^*MaAsLin2 1.18.0*,^41^ and *LinDA 0.2.2*.^42^ We adjusted for confounders and corrected for multiple comparisons using the Benjamini-Hochberg Procedure, and only taxa identified as differentially abundant at *q < *0.05 with all 3 DA methods are reported in the current study.

A microbiome-based index was calculated to summarize the differences in microbes between UC and CD. In line with a previous study,^3^ the index was defined as the natural logarithm of the ratio of overrepresented vs underrepresented taxa in UC compared with CD.

The machine learning procedure is described in detail in Supplementary Methods. In short, machine learning was performed using the Python implementation of *GPBoost 1.5.5*,^43^ nested in a Monte Carlo cross-validation^44^ pipeline. *GPBoost* was chosen due to its support for mixed effects models. The models were assessed using Shapley Additive Explanations (SHAP),^45^ and model performance was measured using area under the receiver operating curve (AUC) values. The pipeline was repeated for 5 sets of features. The first 3 sets were (1) microbiome data, (2) biochemical markers and (3) clinical features, while the latter 2 were (4) a combination of microbiome and biochemical data, or (5) a combination of all 3 data types. Clinical features, such as SCCAI and HBI, were intertwined with diagnostic labels and were therefore not suited for inclusion in the diagnostic models.

## Results

### Study Population

**Figure 1.**
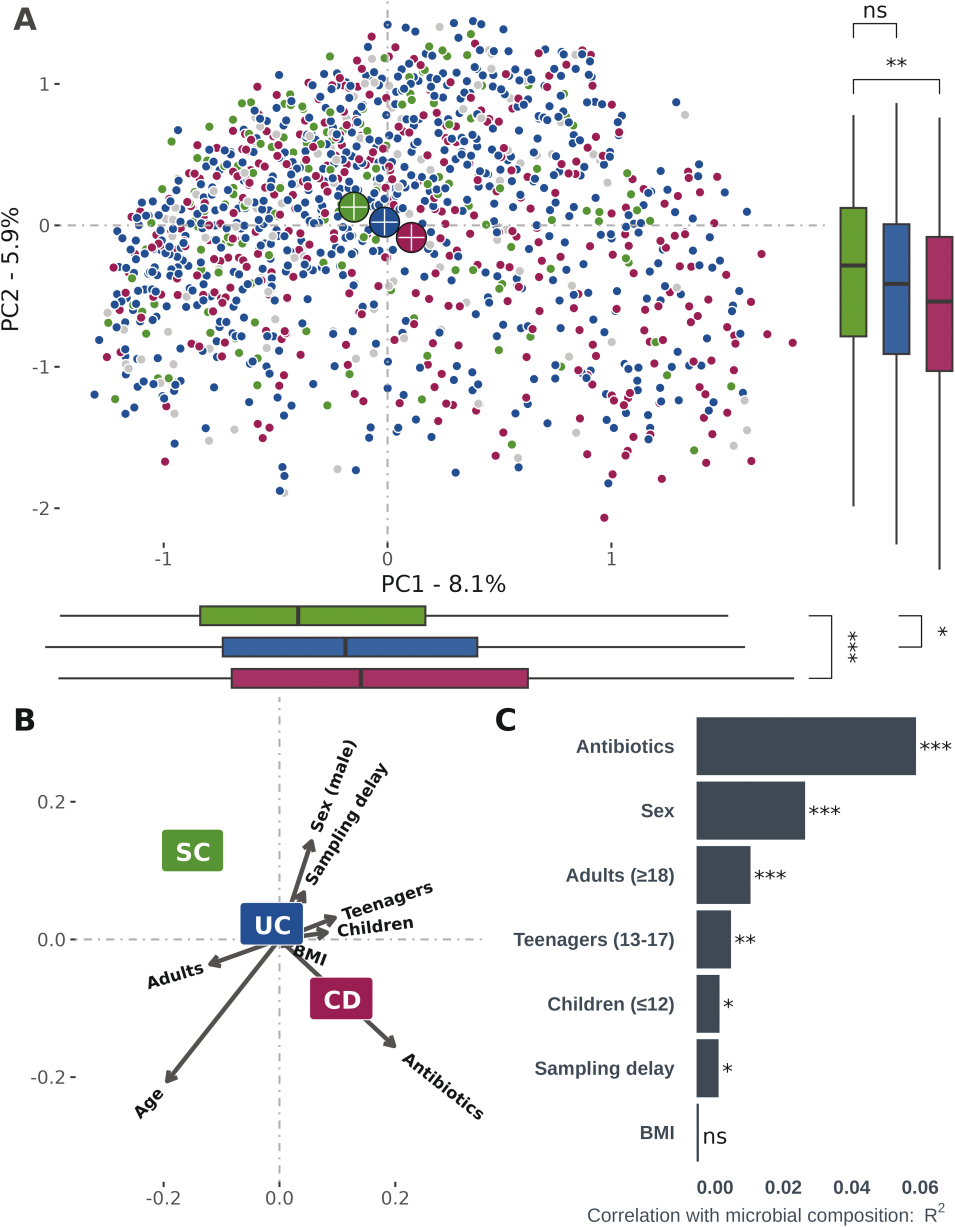
**A**, Beta diversity (Bray-Curtis dissimilarity) plot showing the microbial composition of all participants, colored by disease category (purple = Crohn’s disease [CD], blue = ulcerative colitis [UC], green = symptomatic controls [SC], and gray = miscellaneous categories [IBD-U, suspected IBD colon or suspected IBD small bowel]). The PC1 and PC2 axes denote the amount of variability explained by the principal coordinates. Box plots bordering the x- and y-axes show a significant difference in composition between CD and SC along both PC1 and PC2 (Mann-Whitney, *P* < .001 and *P* < .01, respectively), while there is no such difference between UC and SC (Mann-Whitney, *P* > .05). Adonis/PERMANOVA indicates that there are still compositional differences between these groups (*P* < .0001 for all mentioned comparisons). **B**, Loading plot showing the relationship between bacterial composition (from plot A) and diagnostic groups, age groups, BMI, sex, sampling delay (time from diagnosis to delivered fecal sample), and antibiotics use (within 3 months prior to inclusion). **C,** Barplot demonstrating statistics from the loading plot. Shows that antibiotics use, sex, and age (in the form of adults vs children under 18 years of age) are highly correlated with bacterial composition as visualized in plots A and B (*P* ≤ .001*, R*² > 0.01). To a lesser extent, sampling delay shows some association (*P* < .05*, R*² = 0.007), while BMI is not associated with bacterial composition (*P* = .8, *R*² = 0.0007).

Among the participants in the IBSEN III study, *n* = 1586 (76%) donated fecal samples at inclusion. Following DNA extraction, sequencing, taxonomic filtering, and filtering of low-quality samples, 182 samples were excluded, leaving *n* = 1404 (Table S2). Controlling for diagnosis and sex, we observed that the bacterial composition in children (age < 18 years) was different from that of adults (Figure 1B and C, PERMANOVA *P* = .001, *R*^2^ = 0.005), which in part was driven by a reduced intra-individual (alpha) diversity in children. There was no apparent cutoff between young children (age ≤ 12) and teenagers (aged 13 to 17) (Figure S1A and B), and due to differences in diagnostic criteria and follow-up procedures between pediatric and adult participants, subsequent analyses were conducted within the adult population (≥18 years, *n* = 1229). Therefore, our final population consists of 658 participants with UC, 324 with CD, 36 IBD-U, 67 with suspected IBD, and 144 symptomatic controls (Table 1). For direct comparisons with healthy controls, only samples in the same sequencing library were used (see Methods): CD *n* = 28, UC *n* = 72, symptomatic non-IBD controls *n* = 10, healthy controls *n* = 72.

**Table 1.** Summary of characteristics of the main study population, excluding pediatric participants.

Characteristic	CD *N* = 324	UC *N* = 658	IBD-U *N* = 36	Suspected IBD colon *N* = 26	Suspected IBD small intestine *N* = 41	Symptomatic control *N* = 144	Healthy control *N* = 72	*P*-value^a^
Age, Median (IQR)	40 (27–53)	39 (28–52)	36 (28–52)	38 (30–51)	44 (27–60)	31 (25–41)	42 (38–46)	
Sex, *n* (%)								
Female	194 (60)	321 (49)	20 (56)	14 (54)	27 (66)	73 (51)	35 (49)	
Male	130 (40)	337 (51)	16 (44)	12 (46)	14 (34)	71 (49)	37 (51)	
BMI, Median (IQR)	24.5 (22.1–27.9)	24.8 (22.2–27.9)	24.8 (21.1–27.4)	24.4 (22.1–28.1)	24.9 (22.7–28.7)	24.2 (21.8–27.5)	NA	
Unknown	6	20	3	0	2	5	72	
BSS, Median (IQR)	4 (3–6)	4 (3–5.5)	4 (3–4)	3.75 (3–4)	4 (4–6)	4 (3–6)	NA	
Unknown	169	336	19	16	16	90	72	
Delay^b^, Median (IQR)	23 (8–70)	26 (10–65)	15 (5–50)	20 (2–58)	18 (7–64)	2 (1–5)	NA	
Unknown	10	10	1	2	1	7	72	
Severe course^c^, *n* (%)	51 (17)	56 (9.0)	4 (12)	2 (8.7)	2 (5.9)	0 (0)	NA	
Unknown	17	39	3	3	7	141	72	
Pro/prebioitics^d^, *n* (%)	48 (15)	110 (17)	2 (5.6)	3 (12)	5 (12)	17 (12)	NA	
Unknown	0	0	0	0	0	0	72	
Antibiotics^d^, *n* (%)	40 (12)	53 (8.1)	4 (11)	5 (19)	4 (9.8)	17 (12)	0 (0)	
**Laboratory data**								
F-cal., Median (IQR)	320 (116–1200)	205 (59–859)	262 (80–861)	85 (47–541)	129 (65–263)	47 (29–104)	NA	<.001
Unknown	2	4	1	1	0	7	72	
CRP, Median (IQR)	5 (2–15)	3 (1–6)	3 (1–6)	2 (1–7)	3 (1–5)	1 (1–3)	NA	<.001
Unknown	19	47	3	2	5	1	72	
Hb, Median (IQR)	13 (12–14)	14 (13–15)	14 (13–15)	14 (13–14)	14 (13–15)	14 (13–15)	NA	<.001
Unknown	12	17	2	2	3	2	72	

^a^Kruskal-Wallis rank sum test

^b^Sampling delay, number of days from diagnosis to received fecal samples.

^c^Severe disease course during the first year after inclusion.

^d^Within 3 months preceding inclusion

Abbreviations: CRP, C-reactive protein; F-cal, fecal calprotectin; Hb, hemoglobin.

The characteristics of the final study population mirrored the complete adult IBSEN III population, except for a slightly higher age and proportion of females (Table S3). At the 1-year follow-up, *n* = 51 (17%) of the adult CD and *n* = 56 (9%) of the adult UC population were defined as having a severe disease course, as well as 4 participants with IBD-U. The main reason for classification as severe disease course was hospitalization due to IBD (73 of 112 participants). Fifty-three participants changed IBD classification or diagnosis during the 1-year follow-up, and 18 participants with IBD or suspected IBD were changed to non-IBD-related diagnoses (Table S4).

### Global Microbiome Assessment

Multiple factors were associated with the global microbiome composition, including diagnosis, sex, and age (Figure 1A and B). Antibiotics within 3 months preceding study inclusion, hereafter referred to as just “antibiotics use,” had been prescribed to *n* = 141 (10%) of participants and were associated with reduced alpha diversity (Figure S2). Throughout the result section, analyses were conducted including antibiotics use as a covariate, and additional sensitivity analyses were conducted using only the no-antibiotics population. Sampling delay, that is, the number of days from study inclusion to fecal sample delivery, was associated with increased diversity in UC (*P* = .025 to >.05 depending on cutoff) but not in CD (Table S5 and Figure S3). Stool consistency, as measured by the BSS, was available for a subset (46%) and was associated with both global microbiome composition and alpha diversity, mainly driven by low diversity in those with liquid feces (Figure S4A and B). However, average stool consistency was comparable across all disease groups and endpoints (Mann-Whitney, *P* > .05 for all comparisons). In total, 159 of the participants had used pro- or prebiotics within the last 3 months (Table 1), and we found no effect of the use on the microbiome (diversity and consensus-based DA analysis).

### UC and CD Microbiome Profiles Differ From That of Controls, With Distinct Signatures

**Figure 2.**
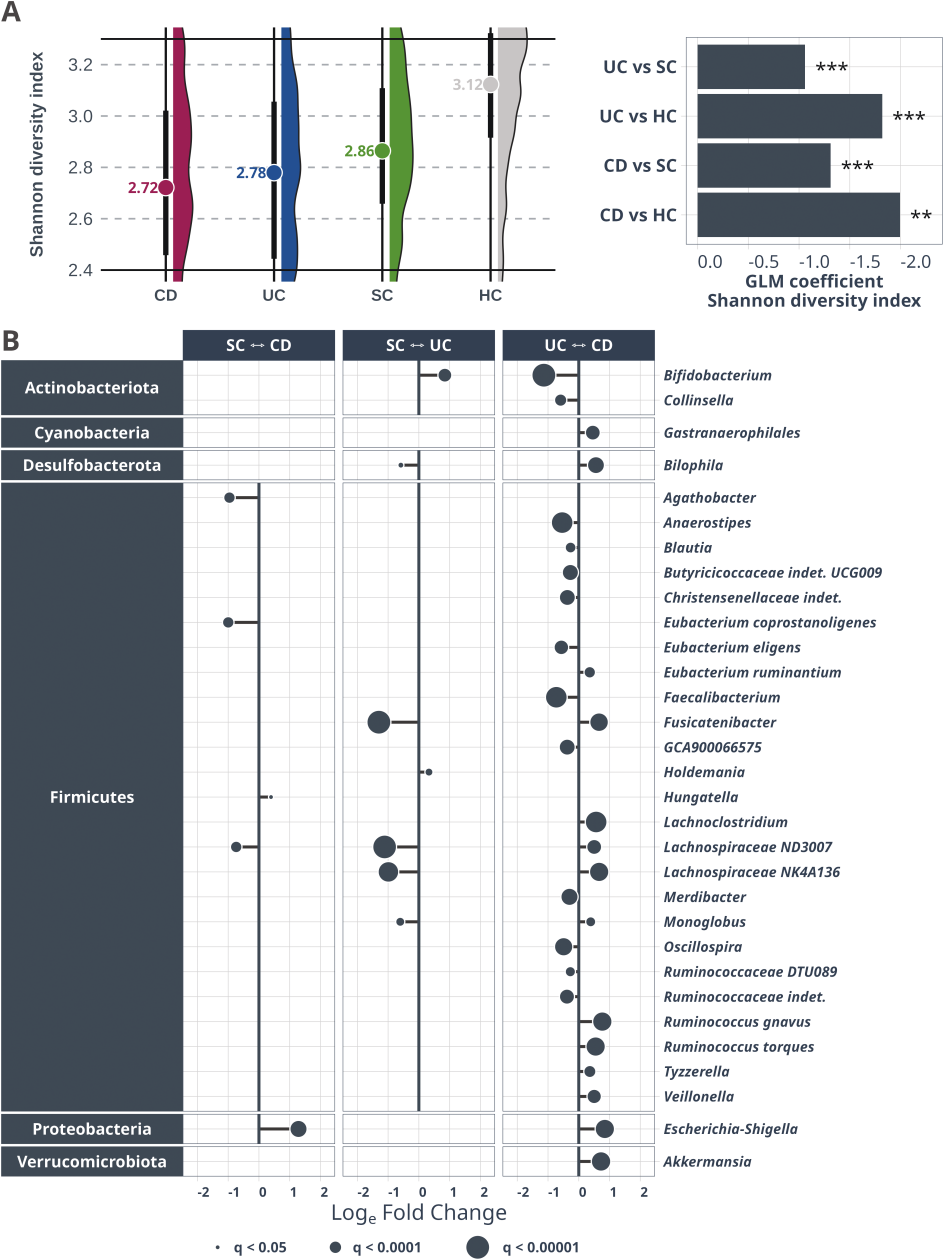
**A,** Alpha diversity plots showing that CD and UC have a reduced diversity as estimated by the Shannon diversity index, compared to symptomatic and healthy controls (SC and HC, respectively). Barplot demonstrates effect size and *P*-value of alpha diversity differences after correcting for age, sex, BMI, and antibiotics in a generalized linear model (GLM). Only samples sequenced together are used for this analysis (see Methods). **B,** Plot visualizing taxa that are differential abundant between the 2 groups by *q* < 0.05. The dot size represents the q value, X-axis demonstrates log(e) fold changes estimated by ANCOM-BC2, but all 3 methods showed similar fold change patterns. Most taxa differing between the diagnostic groups belong to the phylum *Firmicutes*, many of which are significantly decreased in IBD compared to symptomatic controls. Twenty-seven taxa were differentially abundant between UC and CD. CD, Crohn’s disease; IBD, inflammatory bowel disease; UC, ulcerative colitis.

The global gut microbiome composition differed between the IBD groups and both symptomatic and healthy controls (PERMANOVA *P* < .001 for all comparisons), with increased compositional variance within the IBD groups (PERMDISP *P < *.001, Figure S5A and B). The healthy controls had a higher alpha diversity than the symptomatic controls, and both had a higher diversity than IBD, with CD having the lowest diversity overall (Figure 2A). Both compositional and alpha diversity analyses give similar results when repeating the analysis in the non-antibiotic population (Tables S6 and S7).

Compared to symptomatic controls, the microbiome profiles in CD had increased relative abundance of *Escherichia-Shigella* and *Hungatella*, while there were decreased levels of *Agathobacter*, *Eubacterium coprostanoligenes* group, and *Lachnospiraceae ND3007* group (Figure 2B). A mostly non-overlapping set of taxa was different between individuals with UC and symptomatic controls, where UC had increased *Holdemania* and *Bifidobacterium* and decreased *Bilophila*, *Eubacterium ruminantium* group, *Fusicatenibacter, Lachnospiraceae ND3007* group, *Lachnospiraceae NK4A136* group, and *Monoglobus* (Figure 2B). Repeating the analysis within the non-antibiotics population yielded similar results (Table S8).

### Major Microbiome Differences Between UC and CD Summarized in a Microbial Index and Validated in Cohorts From Three Continents

A total of 27 bacterial taxa from 6 different phyla were differentially abundant between UC and CD at *q* < 0.05 (Figure 2B). A UC-CD index was calculated based on the differentially abundant taxa as the log ratio of the sum of taxa increased and taxa decreased (see Methods). Consequently, the index was higher in UC than CD and differentiated between these with an AUC of 0.72 (Table S8). Using the non-antibiotics population, 25 of the 27 taxa remained significant, and an additional taxon was added to the list. Defining a new index based on this list yielded results with overall equal results to that of the original index (Figure S6).

**Figure 3.**
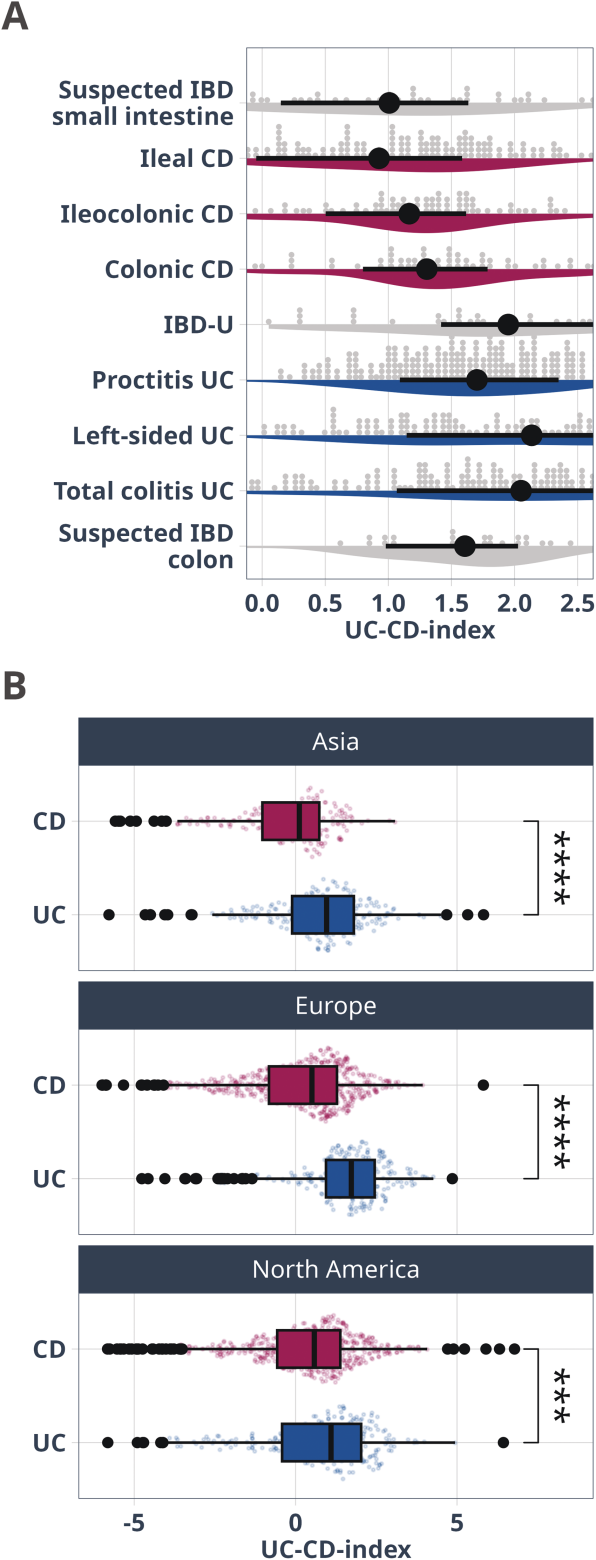
**A**, The UC-CD-index score differs depending on the site of inflammation, defined by CD Montreal localization. Horizontal black lines with dots represent the median and IQR. **B**, Differences between UC and CD in a validation cohort consisting of 36 public datasets. In all 3 examined continents, persons with UC had significantly higher values than CD in the UC-CD index defined in our study. CD, Crohn’s disease; IQR, interquartile range; UC, ulcerative colitis.

To independently validate the ability of the UC-CD index to differentiate UC from CD, we used a collection of publicly available datasets (Table S1). We calculated the UC-CD-index values for samples from North America, Europe, and Asia. In all 3 continents, the index differentiated between UC and CD (AUC 0.59-0.73, Figure 3B, Table S9).

### The Microbial UC-CD-Index Differentiates Phenotypes Across the IBD Continuum

Considering the relationship between disease location and gut microbiome composition, there was a trend suggesting a colon-dependent gradient of increasing UC-CD index values from ileal CD via ileocolonic and colonic CD to UC proctitis, left-sided colitis, and lastly total colitis UC (Figure 3A). In linear models correcting for age, sex, body mass index (BMI) and antibiotics use, there was a higher index in colonic CD compared with ileal CD (*P* = .008) and in UC compared with colonic CD (*P* = .005). Furthermore, repeating the analysis comparing colon CD to UC excluding those with proctitis, we found the index score to remain significantly different (*P* = .0006).

Participants with suspected IBD of the small intestine had UC-CD-index values closest to those with ileal CD, while those with suspected IBD of the colon had index values resembling UC (Figure 3A). Notably, in people who changed classification during follow-up (based on endoscopy and radiology), we observed that those that were initially classified as CD but later reclassified to UC had a microbiome composition closer to CD than UC at baseline, supporting the first diagnosis (Figure S7). Similarly, the microbiome composition of those initially diagnosed as UC and later changed to CD, was more like UC than CD at baseline.

### Microbiome Holds Promises as a Diagnostic Tool When Assessed by Machine Learning

Three different input datasets were used: (1) microbiome profiles, (2) biochemistry data including CRP, F-calprotectin, hemoglobin, ferritin, transferrin, and albumin, and (3) a combination of (1) and (2). Age, sex, BMI, and antibiotics use were included as covariates in all models. Detailed statistics are summarized in Table S10, including comparison to the results when excluding samples exposed to antibiotics.

The biochemistry data had a better ability than microbiome profiles to separate both CD and UC from symptomatic controls, with median AUCs at 0.89 and 0.83, respectively (*P* < .0001, Figure S8A and B, Table S10). Shapley additive explanations analysis was performed to identify the main contributors to the models, suggesting that F-calprotectin was the major differentiator of symptomatic controls from IBD (Figure S8D and E). Shapley additive explanations analyses further showed that high levels of *Bifidobacterium* and low *Fusicatenibacter* and *Lachnospiraceae NK4A136* contributed toward a prediction of UC (Figure S8E), which overlapped with the conventional DA analyses (Figure 2B). For CD, bacterial taxa that were differentially abundant compared with symptomatic controls did not contribute to the machine learning predictions ( Figure S8D).

Regarding the classification of participants with IBD as either CD or UC, microbiome profiles performed significantly better than biochemical markers (Figure S8C, *P* < .0001). We validated the microbiome-based models in the validation cohort (AUC 0.64-0.76, Table S11). Among several variables, a high relative abundance of *Bifidobacterium* was indicative of UC, while an increased *Ruminococcus torques* group was associated with CD (Figure S8F). There was a large overlap of taxa differentiating UC vs CD as evaluated by DA testing and machine learning (Figure 2B and Figure S8F). The more clinically relevant comparison of UC with only left-sided or total colitis vs colonic CD was harder to predict, with a significantly lowered AUC (0.73 → 0.67) and a low positive predictive value (0.50 −> 0.25, Table S10). However, microbiome profiles remained the strongest predictor compared to biochemical markers (*P* < .0001).

### Fecal Microbiome Composition Predicts a Severe Disease Course in UC

**Figure 4.**
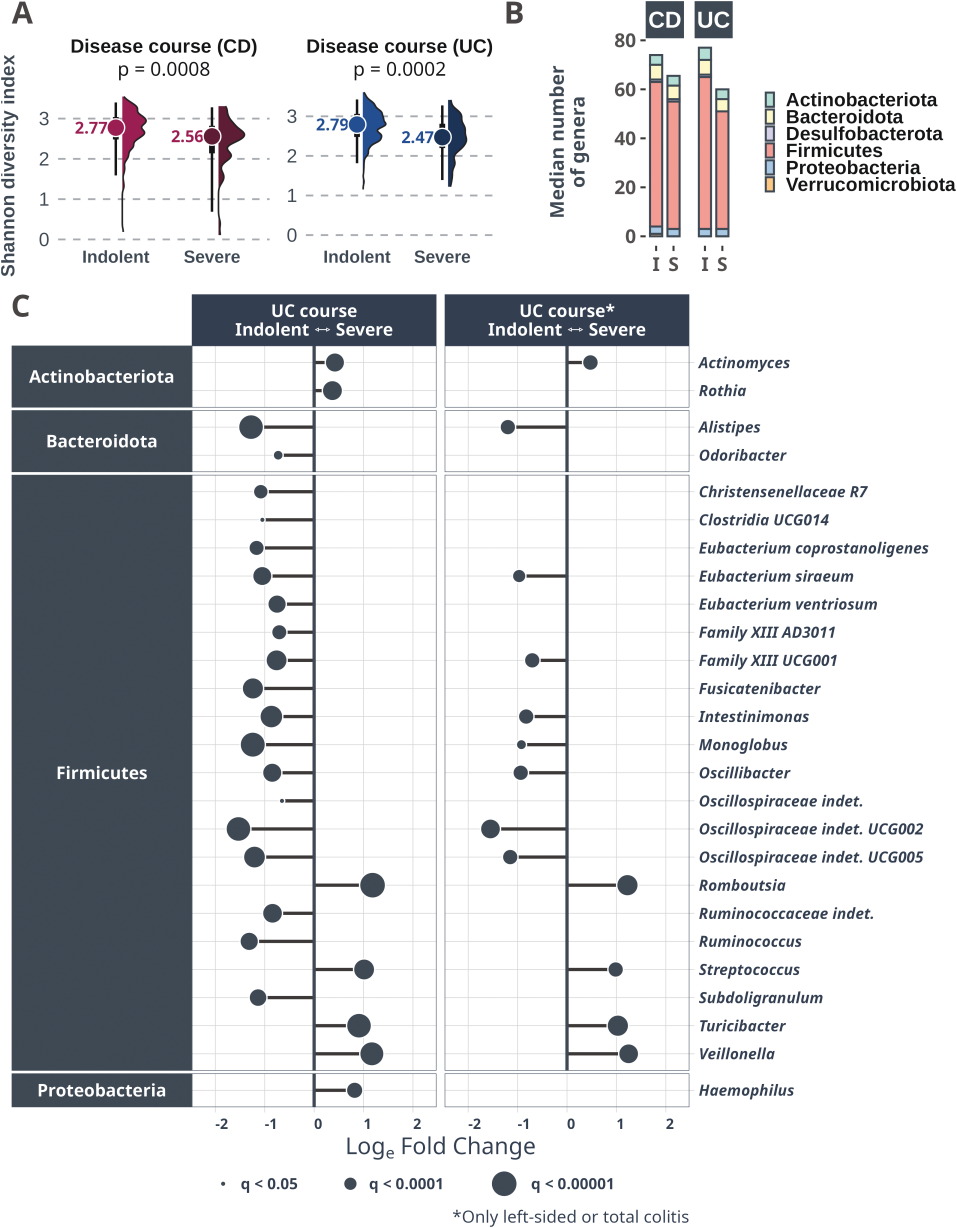
**A**, Alpha diversity plots demonstrating a reduced diversity as measured by the Shannon diversity index in those patients with a future severe disease course in CD and UC, respectively. **B,** Bar plot showing average numbers of different genera, demonstrating that the major factor affecting the loss of diversity is the phylum *Firmicutes* (which contains the class *Clostridia*). **(C)** Differential abundance plot showcasing the results from the 3 DA methods (ANCOM-BC2, LinDA, and MaAsLin2). Only taxa with FDR-adjusted *P*-values (*q*) less than .05 in all 3 methods are included. The dot size represents the *q* value, X-axis demonstrates log(e) fold changes estimated by ANCOM-BC2, but all 3 methods showed similar fold change patterns. Most taxa differing between the diagnostic groups belong to the class *Clostridia*, many of which are significantly decreased in UC patients with a future severe disease course compared to those with an indolent disease course. Twenty-six taxa were differentially abundant between patients with severe and indolent disease courses. CD, Crohn’s disease; UC, ulcerative colitis.

Alpha diversity was reduced at baseline in persons with IBD who experienced a severe disease course over the first year, compared with those with an indolent disease course, both for IBD and when analyzing CD and UC separately (Figure 4A). In UC, 26 taxa were differentially abundant between individuals with severe and indolent disease course (*q < *0.05, Figure 4B).

**Figure 5.**
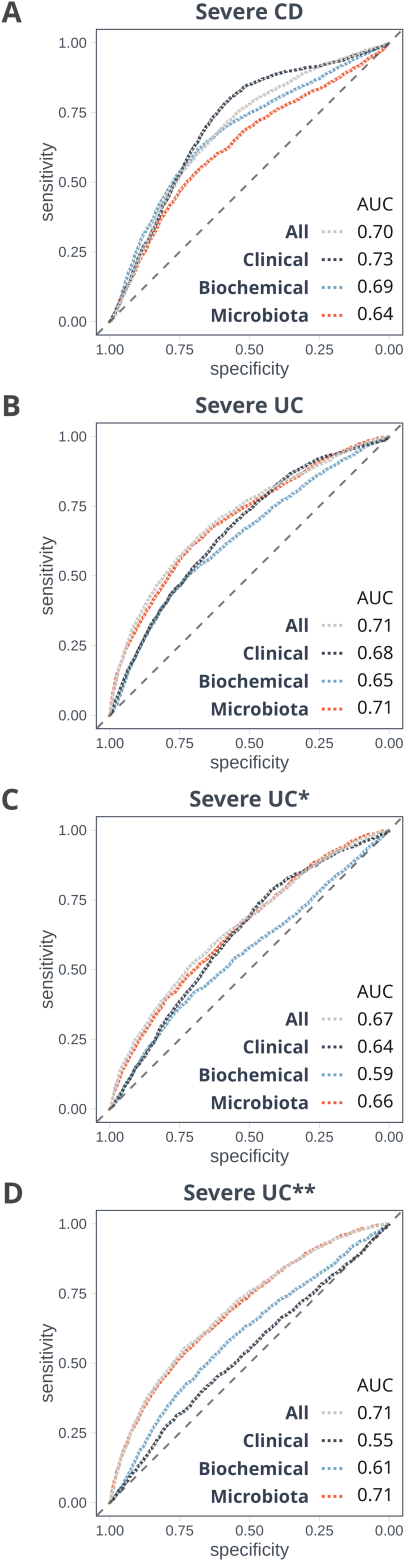
**A, B, C, D,** AUC curves showing the predictive capacity of 3 different sets of variables for classifying a future severe disease course in CD and UC, respectively. In CD, the variable set “Clinical” scored significantly better than microbiome data (*P* < .0001). The opposite was true in UC, where the microbiome held the best predictive capacity of the 2 (*P* < .0001). When excluding patients admitted with a severe baseline Mayo endoscopic subscore with left-sided to total colitis, the microbiome was still the best performer, but no longer significantly better than “Clinical.” *Excluding severe baseline Mayo score with left-sided to total colitis; **Only left-sided or total colitis. AUC, area under the receiver operating curve; CD, Crohn’s disease; UC, ulcerative colitis.

Machine learning models were used to compare the prognostic value of microbiome profiles with the biochemical markers, endoscopic data, and clinical disease activity at baseline (Table S10). Microbiome profiles significantly outperformed biochemical markers and clinical features as predictors of a severe disease course in UC (median AUC 0.71, *P* < .001, Figure 5B and Table S10). Neither biochemical markers nor clinical scores provided any additional predictive value on top of microbiome profiles (Figure 5B). Low levels of *Lachnospiraceae GCA-900066575* and high levels of *Streptococcus* and *Romboutsia* were the main indicators of a severe disease course (Figure S9E).

Among individuals with UC experiencing a severe disease course during the first year, *n* = 51 (75%) had a mild disease at inclusion (proctitis and/or Mayo endoscopic subscore 0-2). In this group, the fecal microbiome remained the strongest individual predictor (AUC = 0.66), closely followed by the clinical scores (AUC = 0.64, Figure 5C). High relative abundance of *Streptococcus* and deficiencies of *Lachnospiraceae GCA-900066575*, *Eubacterium siraeum* group, *Monoglobus*, and *Flavonifractor* were all predictive of a future severe disease course (Figure S9F).

Within the CD group, there were no specific genera associated with a severe disease course according to our criteria. When aiming to predict a severe disease course in CD, biochemical markers and clinical features outperformed the microbiome profiles (*P* < .0001, Figure 5A), with F-calprotectin being the most important predictor overall (Figure S9D).

## Discussion

In this large Norwegian population-based inception cohort of people with suspected IBD, the fecal microbiome composition showed a potential benefit as a noninvasive tool to identify characteristics and severity of IBD, although it was less valuable than biochemical disease markers in separating IBD from symptomatic non-IBD. The fecal microbiome separated UC from CD, which could be validated in international cohorts. We observed a gradient of fecal microbial alterations explained by disease location, with ileal CD and colonic CD being as distinct from each other as colonic CD is from UC. While the consistent ability of the UC-CD index to separate the IBD subtypes suggests that these have distinct microbiome profiles, the observation that people changing classification have microbiome characteristics similar to the initial diagnosis may imply that microbiome will not be helpful to differentiate between difficult cases. The microbiome profile at baseline in UC was able to predict a severe disease course at 1 year with higher precision than the investigated biochemical or clinical parameters, also in individuals with mild disease at inclusion. Overall, the clinical application of the fecal microbiome profile is relevant for disease classification and prediction of prognosis beyond the severity of inflammation, with implications for clinical care and choice of therapy.

Alpha diversity was lower in CD than UC, and the microbial composition diverged more from symptomatic controls in CD than in UC. This is in line with studies using healthy individuals as a control group, where CD diverges most from a healthy state.^1,46^ There are few IBD microbiome studies using symptomatic controls for comparison. However, Pascal et al.^46^ described a microbial signature with a similar ability to separate CD from UC and symptomatic controls, mirroring our machine learning results. In our study, biochemical parameters driven by F-calprotectin exhibited superior predictive performance compared to microbiome profiles in diagnosing IBD. Despite larger differences in microbiome composition compared with symptomatic non-IBD, the microbiome profiles did not contribute substantially to improved prediction on top of biochemistry in CD, while some improvement was seen in UC. It is possible that a proportion of our symptomatic controls had irritable bowel syndrome, which is also associated with dysbiosis.^47,48^ In earlier studies, comparisons between people with IBD and healthy controls may have resulted in overinflated diagnostic estimates. Therefore, diagnostic tools should be developed in the context of the clinical problem, that is, utilizing people with similar symptoms but without IBD as the control group, as done in the current study.

Microbiome profiles outperformed biochemical markers in distinguishing UC from CD. This held true even when restricted to colonic CD and left-sided/total colitis in UC, though with a low positive predictive value which decreased from 0.50 to 0.25 (Table S10). Previous studies report varied microbiome performance for within-IBD classification,^1,38,46^ but study populations in microbiome research are often too small for meaningful conclusions.^15^ Recently, Vestergaard et al.^38^ defined microbial differences between CD and UC using a similar analysis pipeline as ours with the requirement of a consensus of 3 different statistical tools. They combined and reanalyzed public datasets from 45 different worldwide cohorts, with only 7 statistically significant taxa differentiating UC and CD in common with our study.^38^ This could in part be explained by geographically dependent differences.^49,50^ However, by summarizing our findings as a UC-CD index and calculating this index with the international data used by Vestergaard et al., we were able to validate our findings on 3 continents. This suggests that geographically independent patterns exist, opening the space for microbiome-based clinical tools with wider applications.

Location of inflammation is an important clinical feature^51^ and is more stable over time than disease activity.^52^ The microbiome differences between CD and UC were associated with location of disease, as the UC-CD index showed a colon-dependent gradient where colonic CD represented a midpoint between ileal CD and UC. The index score of colonic CD was closest to proctitis; however, these are rather easily discriminated from colonic CD in the clinic. Distinguishing left-sided or total colitis from colonic CD is clinically more difficult, and here the index yielded significantly different scores. The observation of a gradient-like index is in line with previous genetic and multi-omics studies, which describe IBD as a continuum.^51,52^ Cleynen et al.^52^ suggested that the spectrum of IBD can be most accurately represented by 3 distinct categories, ileal CD, colonic CD, and UC. A molecular approach to disease classification could be more informative than the traditional UC-CD dichotomy in therapy decision-making, since it may provide a more nuanced picture and precise measurements of underlying pathophysiological mechanisms. Still, this does not imply that microbiome composition is the primary driver of disease. Approximately one in 20 participants with IBD changed diagnosis during the first year (Table S4), which is common.^53^ For these, the microbiome profile at baseline was largely consistent with their original diagnosis. People changing from UC at the time of diagnosis to CD at the 1-year follow-up had a baseline microbiome resembling UC and vice versa. The microbiome composition may therefore reflect the current state of disease (eg, inflammation and location or other unknown characteristics). These observations provide a rationale for studies of microbiome dynamics during disease follow-up to understand this phenomenon in more detail.

A severe disease course during the first year after diagnosis was associated with a less diverse baseline microbiome composition. This finding is in line with a previous report on pediatric UC.^54^ The ability to predict severe disease course was strongest in UC, and in line with reduced diversity, it depended mainly on the depletion of multiple taxa. Many of these reduced taxa belonged to the class *Clostridia*, which is interesting in the context of the “oxygen hypothesis,” where increased oxygen in the intestine in active IBD is proposed to reduce the abundance of the obligate anaerobe short-chain fatty acid-producing *Clostridia*.^55,56^ Short-chain fatty acids are microbial-derived metabolites essential for intestinal homeostasis, commonly depleted in IBD.^11,57,58^ Although the observational design of our study makes it difficult to reach causal conclusions, the clinical benefit of fecal microbiome transplantation in some studies—mainly in UC—suggests that the microbial composition is relevant for disease activity.^4,59,60^ The subgroup with depleted health-promoting bacteria represents a particularly interesting target for further fecal microbiome transplant experiments.

Microbiome profiles remained the strongest predictor of future severe disease course in UC, even after the exclusion of the subgroup with left-sided/total colitis with a Mayo endoscopic subscore of 3 at baseline from the analysis. Adding both clinical and biochemical parameters did not increase the performance, suggesting that microbiome composition can provide predictive power not attained by simply measuring inflammation parameters. In terms of which data category was most informative, these results were unchanged when excluding samples with antibiotics exposure from the models. However, the microbiome-based models had only a modest performance (AUC = 0.66, or 0.68 in the no-antibiotics population), and the importance of the microbiome as a prognostic factor is still unclear. Nevertheless, these findings could motivate further work to define the relevant pathogenic mechanisms so they can be measured and targeted therapeutically.

The major strength of this study is the use of a large inception cohort with a population-based multicenter approach including participants based on suspicion of IBD. Baseline samples were analyzed to assess the clinical relevance of early fecal microbiome profiling. A key advantage is the conservative analysis approach requiring the consensus of 3 different statistical tools as recommended by Nearing et al.^39^ Additionally, we applied machine learning methods to quantify diagnostic capabilities, with a secondary explanatory procedure to identify the determinants of the predictions. The latter is critical in the development of microbiome tests aimed to improve clinical decision-making. Among participants diagnosed with IBD, the median time between diagnosis and arrival of fecal sample was 18 days. This represents a potential limitation, since we do not have complete control of the day of treatment initiation vs the day of sampling. However, sampling delay was included as a covariate. We did not adjust for stool consistency in our study, though this information was available as patient-reported BSS values for about half of the samples. Bristol Stool Scale was associated with both bacterial composition and overall diversity scores. However, no disease groups differed in terms of BSS, and neither did those with a severe disease course when compared to those with an indolent course. It would nonetheless be of interest for future studies to obtain complete BSS data so that its role as a confounder in microbiome studies on IBD can be explored in more detail. We also included in the main analysis people who were treated with antibiotics before the IBD diagnosis, which would make the results more generalizable. Importantly, subanalyses without the antibiotics-treated individuals were highly similar.

The overall disease severity was mild in the present population, with only 10%-17% meeting the criteria for a severe disease course, which may limit the power of some analyses given the stringency of our statistical approach. This could partly explain the lack of microbial associations when looking at CD with a severe disease course (see Supplementary Discussion), but highlights the strength of the identified microbial associations with severe disease course in UC. The estimated performance of microbial profiles for prediction has an uncertainty caused by inherent limitations in 16S rRNA sequencing, and many newer studies use metagenomic shotgun sequencing instead. Despite the limitation of using 16S rRNA sequencing for microbiome profiling in obtaining species and strain level resolution, 16S-based prediction model performance is shown to be similar between metagenomic shotgun and 16S-based datasets.^61–63^ Another limitation is that we did not adjust for dietary patterns, which is a major factor that may shape the microbial composition. Information about early life events was not available in our data and was therefore not considered. Finally, our study population is from only one geographical area. However, the multi-national validation of some of our findings suggests global generalizability.

In conclusion, fecal microbiome profiles may show promise as a noninvasive tool for the classification of IBD subtypes, prior to and in concert with established endoscopic and histological diagnostic criteria. Additionally, we show interesting signals in microbiome profiles linked to future disease course in UC. These observations may guide the next steps when developing microbiome-based diagnostic tools and may be an important step toward early detection of individuals with UC at high risk of experiencing a severe disease course.

## Supplementary Data

Supplementary data is available at *Inflammatory Bowel Diseases* online. Additional supplementary figures not mentioned in the main article provide details on machine learning results for severe disease course (S10), rarefaction analysis (S11) and machine learning noise analysis on synthetic data (S12).

izaf060_suppl_Supplementary_Methods

izaf060_suppl_Supplementary_Tables_2-11

izaf060_suppl_Supplementary_Figures_1-9

izaf060_suppl_Supplementary_Tables_1

izaf060_suppl_Supplementary_Tables_10

izaf060_suppl_Supplementary_Materials

## Data Availability

Available upon reasonable request.
