# Supplementary material for: Fecal Microbiome Reflects Disease State and Prognosis in Inflammatory Bowel Disease in an Adult Population-Based Inception Cohort
Source: Inflamm Bowel Dis. 2025 Apr 25;31(8):2066–80. doi: 10.1093/ibd/izaf060 (PMC12491950; doi:10.1093/ibd/izaf060)
Supplement: izaf060_suppl_Supplementary_Methods [file izaf060_suppl_supplementary_methods.docx]

**Fecal microbiome reflects disease state and prognosis in inflammatory bowel disease in an adult population-based inception cohort**

**SUPPLEMENTARY METHODS AND DISCUSSION**

### Sample processing and sequencing

The fecal samples were thawed and transferred to 0.70 mm Garnet Bead tubes (Dianova, Hamburg, Germany) containing 1.1 mL InhibitEX lysis buffer, before being homogenized using SpeedMill PLUS (Analytik Jena, Jena, Germany) for 45 seconds at 50 Hz. The samples were then heated to 95 °C for 5 minutes before proceeding with DNA extraction on the QIAcube system (Qiagen) using the QIAamp DNA Stool Mini Kit (Qiagen) as per the manufacturer’s protocol.

The sequencing libraries were prepared according to standard protocol [1]. As a summary, we ran PCR amplifications of the V3-V4 region of the 16S rRNA gene using Phusion High-Fidelity PCR Master Mix with HF buffer (Thermo Fisher Scientific), utilizing dual-indexed universal primers 319F (forward) and 806R (reverse). The PCR amplicons were then cleaned and normalized using the SequalPrep Normalization Plate Kit (Thermo Fisher Scientific), before being quality controlled with both Agilent Bioanalyzer (Agilent Technologies) and Kapa Library Quantification Kit (Kapa Biosystems). After the quality control, the normalized amplicons were pooled and sequenced with the Illumina MiSeq platform and v3 kit (Illumina) at the Norwegian Sequencing Centre in Oslo.

DNA was isolated in two batches. and sequenced in 8 different 16S rRNA amplicon sequencing libraries. Samples yielding less than 10 000 reads were re-sequenced.

### Bioinformatics processing

The sequenced amplicons were filtered for Illumina universal adapters and PhiX sequences using *bbduk* *38.92* [2] (adapters filtered with *k=23 hdist=1 tbo cf=TRUE ftm=5*, PhiX filtered with *k=31, hdist=1*). The filtered reads were demultiplexed according to their dual-indexed universal primers before the primers, heterogeneity spacers and indices were trimmed using *cutadapt* *3.4* [3] (demultiplexed with *-e 1 –no-indels –overlap 12*, trimming with -*e 0.1 –overlap 20 –m 250*). The paired reads were then merged and quality trimmed using *bbmerge 38.92* [4] (filtered and merged with *qtrim=rl trimq=15 maxlength=440 mininsert=390*).

The merged reads were then denoised to amplicon sequence variants (ASVs; taxonomic units) using *Deblur* [5] as implemented in *QIIME2 2021.4* [6]. ASVs detected as singletons (i.e. occurring in only one sample) were removed before the remaining reads were taxonomically classified using a naïve Bayes classifier [7]. The classifier was pre-trained on the V3-V4 region of a pre-clustered version (99% sequence similarity) of the SILVA 138 database [8] (SILVA reads prepared for training with RESCRIPt [24] *–p-mode “super”*).

Based on 99 negative controls from various stages of the DNA isolation and library preparation steps a contaminant profile was defined as taxa occurring in >50% of controls, and the following bacteria deemed as contaminants were removed from the dataset: *Pseudomonas, Stenotrophomonas, Yerseniaceae* sp., *Serratia, Burkholderia-Caballeronia-Paraburkholderia* and *Alcaligenaceae.* A taxon classified as *Chloroplast* occurred more rarely than the threshold but was removed due to its likely plant food origin. The remaining ASVs were then collapsed to the genus level and exported from *QIIME2* for subsequent analysis and modeling. All further analysis was done in R *4.2.3* [9] and visualized with the R package *ggplot2 3.4.1* [10], unless stated otherwise.

Rare taxa occurring in less than 10%of samples were filtered out, before samples with less than 9500 reads were excluded (cutoff based on rarefaction curve analysis (Supp. Fig 11)). After exclusion of rare taxa and low-read samples, a rarefied version of the data was calculated based on the common lowest read count (9506 reads).

### Bioinformatics analysis and statistics

Confounders were assessed by examining their relationship with bacterial diversity and composition. The examined confounders include age, sex, BMI, antibiotics use (within 3 months prior to study inclusion) and sampling delay (time from diagnosis to fecal sample received at biobank), as well as technical confounders including sequencing batch (hereafter called “library”), DNA extraction batch and recruitment site as subjects were recruited from 20 different hospitals. Library was corrected for as a random effect in mixed model designs, whereas DNA extraction batch and recruitment site were not associated with the microbiome and were not included in further analyses. Bristol stool scale (BSS) evaluations existed for a subset of samples and were included as a covariate only in specific analyses.

Variables related to biochemistry with spurious missing values were imputed using K-means based median imputation, in order to avoid loss of data during types of analyses that could not handle missing values, such as envfit.

Intra-individual (alpha) diversity indices (Shannon diversity index and the total number of observed taxa per sample) were calculated on the rarefied dataset. Beta diversity, the comparative microbial compositions between samples, were examined with the R-package *vegan 2.6-4* [11], using Bray-Curtis dissimilarities on rarefied bacterial data normalized with frequency counts of positive occurrences [12]. The relationship between bacterial composition and other variables was calculated with the *envfit* function of *vegan* (permutations = 999).

For comparisons of the relative abundance of different taxa (differential abundance testing), we used a consensus-based approach as recommended by Nearing *et al.* [13]. Three methods were chosen based on their ability to account for sequencing depth heterogeneity due to batch effects: *ANCOM-BC2 2.0.3* [14], *MaAsLin2 1.18.0* [15] and *LinDA 0.2.2* [16]. All methods corrected for age, sex, BMI, antibiotics and sampling delay. In MaAsLin2 and LinDA, library was controlled for as a random effect, while ANCOM-BC2 implicitly corrects for differences in sequencing depth on a sample-wise level [14]. Only taxa identified as differentially abundant at *q* < 0.05 with all three methods are reported in this paper.

A microbiome-based index was calculated to summarize the differences in microbes between UC and CD. In line with a previous study [17], the index was defined as the logarithm of the ratio of the sum of taxa overrepresented in one of the conditions and the sum of the taxa underrepresented in the same condition.

Machine learning was performed using GPBoost, a method that allows for the combination of tree-boosting and mixed effects models [18]. To compare the predictive capability of the microbiome with biochemical markers and clinical features, an iterative design based on Monte Carlo cross-validation [19] was employed using the same 100 0.66/0.33 train/test splits for five different sets of features. The first three sets were (1) microbiome data, (2) biochemical markers and (3) clinical features, while the latter two were (4) a combination of microbiome and biochemical data, or (5) a combination of all three data types. Clinical features were used to define diagnostic labels and were therefore not included in diagnosis-related models. Hyperparameter tuning was done per combination of feature set and outcome variables using a grid search script that only utilized the training split of each feature set. To explore the predictive roles of the input variables, SHapley Additive exPlanations - referred to as SHAP values - were calculated for each machine learning model [20]. To simplify interpretation of SHAP value visualizations, feature values were transformed in plots so that all above-median values were equal to 1, and all below-median values were equal to -1. The total performance of each set was measured per iteration using the area under a receiver operating characteristic (ROC) curve, referred to as the AUC, and the median AUC of the 100 models are reported per feature set.

### DISCUSSION

### Size of investigated groups brings technical limitations to machine learning

The amount of participants classified with a severe disease course was relatively low in regard to the amount of taxonomic features investigated. In particular, we saw that the machine learning models performed inadequately in the case of a severe disease course among CD patients, and they were unable to effectively filter out irrelevant features when biochemical, clinical and microbial data were combined (Fig 2D and 3D). We show that this is a power issue with an experimental dataset containing varying amounts of noisy variables in three different datasets with n=20, 50 or 500 cases and n=100, 200 or 500 controls (Supp. Fig 12). With this simulated data, the machine learning approach managed to effectively utilize only the 5 predictive features in the presence of a large amount of random noise (995 noisy variables) only when n=500/500 cases/controls. When n=50/200 cases/controls (similar to our CD population), machine learning was negatively affected by the presence of noisy variables (median AUC 0.63 versus 0.78, *p* < 0.0001). This power problem might explain why some ML models using combined datasets performed worse than single source datasets (i.e. microbiome, biochemical or clinical alone).

REFERENCES

1. Fadrosh, D.W., et al., *An improved dual-indexing approach for multiplexed 16S rRNA gene sequencing on the Illumina MiSeq platform.* Microbiome, 2014. **2**(1): p. 6.

2. Bushnell, B., *BBTools software package.* URL <http://sourceforge>. net/projects/bbmap, 2014. **578**: p. 579.

3. Martin, M., *Cutadapt removes adapter sequences from high-throughput sequencing reads.* EMBnet. journal, 2011. **17**(1): p. 10-12.

4. Bushnell, B., J. Rood, and E. Singer, *BBMerge–accurate paired shotgun read merging via overlap.* PloS one, 2017. **12**(10): p. e0185056.

5. Amir, A., et al., *Deblur rapidly resolves single-nucleotide community sequence patterns.* MSystems, 2017. **2**(2): p. e00191-16.

6. Bolyen, E., et al., *Reproducible, interactive, scalable and extensible microbiome data science using QIIME 2.* Nature biotechnology, 2019. **37**(8): p. 852-857.

7. Bokulich, N.A., et al., *Optimizing taxonomic classification of marker-gene amplicon sequences with QIIME 2’s q2-feature-classifier plugin.* Microbiome, 2018. **6**(1): p. 1-17.

8. Quast, C., et al., *The SILVA ribosomal RNA gene database project: improved data processing and web-based tools.* Nucleic acids research, 2012. **41**(D1): p. D590-D596.

9. Team, R.C., *R: A language and environment for statistical computing; 2021*. 2021, R Foundation for Statistical Computing: Vienna, Austria.

10. Wickham, H., *ggplot2.* Wiley interdisciplinary reviews: computational statistics, 2011. **3**(2): p. 180-185.

11. Oksanen, J., et al., *vegan: Community Ecology Package. R package version 2.5-7. 2020*. 2021.

12. Oksanen, J., *Ordination of boreal heath-like vegetation with principal component analysis, correspondence analysis and multidimensional scaling.* Vegetatio, 1983. **52**(3): p. 181-189.

13. Nearing, J.T., et al., *Microbiome differential abundance methods produce different results across 38 datasets.* Nat Commun, 2022. **13**(1): p. 342.

14. Lin, H. and S.D. Peddada, *Analysis of compositions of microbiomes with bias correction.* Nature communications, 2020. **11**(1): p. 1-11.

15. Mallick, H., et al., *Multivariable association discovery in population-scale meta-omics studies.* PLoS Comput Biol, 2021. **17**(11): p. e1009442.

16. Zhou, H., et al., *LinDA: linear models for differential abundance analysis of microbiome compositional data.* Genome Biol, 2022. **23**(1): p. 95.

17. Gevers, D., et al., *The treatment-naive microbiome in new-onset Crohn’s disease.* Cell host & microbe, 2014. **15**(3): p. 382-392.

18. Sigrist, F., *Latent Gaussian Model Boosting.* IEEE Transactions on Pattern Analysis and Machine Intelligence, 2023. **45**(2): p. 1894-1905.

19. Picard, R.R. and R.D. Cook, *Cross-Validation of Regression Models.* Journal of the American Statistical Association, 1984. **79**(387): p. 575-583.

20. Lundberg, S.M. and S.-I. Lee, *A unified approach to interpreting model predictions.* Advances in neural information processing systems, 2017. **30**.
