# Supplementary material for: Fecal Microbiome Reflects Disease State and Prognosis in Inflammatory Bowel Disease in an Adult Population-Based Inception Cohort
Source: Inflamm Bowel Dis. 2025 Apr 25;31(8):2066–80. doi: 10.1093/ibd/izaf060 (PMC12491950; doi:10.1093/ibd/izaf060)
Supplement: izaf060_suppl_Supplementary_Tables_2-11 [file izaf060_suppl_supplementary_tables_2-11.docx]

SUPPLEMENTARY TABLES

*[Supplementary Table 1 is attached in a separate pdf]*

Supplementary Table 1. Summary and description of all datasets used as a validation cohort by the current study.

| **Characteristic** | **CD**,  N=404 | **UC**,  N=707 | **IBD-U**,  N=47 | **Suspected**  **IBD colon**,  N=31 | **Suspected IBD small intestine**,  N=49 | **Symptomatic**  **control**,  N=166 | **Healthy**  **control**, N=72 | **p-**  **value***^*^* |
| --- | --- | --- | --- | --- | --- | --- | --- | --- |
|  |  |  |  |  |  |  |  |  |
| Age, Median (IQR) | 33 (20-48) | 36 (26-51) | 30 (21-46) | 33 (24 - 48) | 35 (24-58) | 29 (22-38) | 42 (39-45) |  |
| Pediatric, n (%) | 80 (20) | 49 (6.9) | 11 (23) | 5 (16) | 8 (16) | 22 (13) | 0 (0) |  |
| Sex, n (%) |  |  |  |  |  |  |  |  |
| male | 176 (44) | 358 (51) | 22 (47) | 15 (48) | 19 (39) | 85 (51) | 37 (51) |  |
| BMI, Median (IQR) | 23.4 (20.4-27.2) | 24.5 (21.8-27.7) | 23.8 (20.8-26.8) | 23.9 (20.8-26.5) | 24.8 (21.9-26.9) | 23.7 (21.1-26.6) | NA |  |
| Unknown | 6 | 22 | 5 | 0 | 2 | 5 | 72 |  |
| BSS, Median (IQR) | 4 (3-6) | 4 (3-5) | 4 (3-5) | 4 (3-4) | 4 (3-6) | 4 (3-4) | NA |  |
| Unknown | 223 | 365 | 26 | 20 | 22 | 98 | 72 |  |
| Delay^**^, Median (IQR) | 16 (7-53) | 24 (9-63) | 15 (6-36) | 12 (3-31) | 14 (6-47) | 2 (1-9) | NA |  |
| Unknown | 11 | 11 | 1 | 2 | 1 | 9 | 72 |  |
| Antibiotics^***^, n (%) | 49 (12) | 58 (8.2) | 7 (15) | 5 (16) | 5 (10) | 17 (10) | 0 (0) |  |
| Severe Course, n (%) | 65 (17) | 68 (10) | 5 (12) | 2 (7.7) | 2 (4.9) | 0 (0) | NA |  |
| Unknown | 18 | 40 | 5 | 5 | 8 | 160 | 72 |  |
| **Laboratory data** |  |  |  |  |  |  |  |  |
| F-cal.^****^, Median (IQR) | 320 (116-1,191) | 205 (59-849) | 262 (82-830) | 85 (47-501) | 129 (65-263) | 47 (29-104) | NA | <0.001 |
| Unknown | 2 | 4 | 1 | 1 | 0 | 7 | 72 |  |
| CRP, Median (IQR) | 5 (2-15) | 3 (1-6) | 3 (1-6) | 2 (1-7) | 3 (1-5) | 1 (1-3) | NA | <0.001 |
| Unknown | 19 | 47 | 3 | 2 | 5 | 1 | 72 |  |
| Hb, Median (IQR) | 13 (12-14) | 14 (13-15) | 14 (13-15) | 14 (13-14) | 14 (13-15) | 14 (13-15) | NA | <0.001 |
| Unknown | 12 | 17 | 2 | 2 | 3 | 2 | 72 |  |


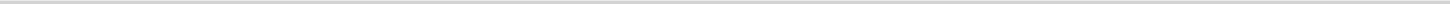


^*^Kruskal-Wallis rank sum test, ^**^Sampling delay, ^***^Antibiotics within 3 months preceding study inclusion, ^****^Fecal calprotectin

Supplementary Table 2. Summary of the complete population, before exclusion of pediatric participants are.

| *Diagnosis* | *n Our study* | *% of IBD-population (1158)* | *n IBSEN III (2085)* | *% of IBD population in IBSEN III* | *% females Our study* | *% females IBSEN III* | *Median age Our study* | *Median age IBSEN III* |
| --- | --- | --- | --- | --- | --- | --- | --- | --- |
| *Total* | *1229* | *-* | *1888* | *-* | *-* | *-* | *-* | *-* |
| *CD* | *324* | *32%* | *509* | *32.5%* | *60%* | *54%* | *40* | *37* |
| *UC* | *658* | *64.5%* | *1003* | *64%* | *49%* | *45%* | *39* | *36* |
| *IBD-U* | *36* | *3.5%* | *52* | *3.5%* | *56%* | *50%* | *36* | *35* |
| *SC** | *144* | *-* | *221* | *-* | *51%* | *54%* | *31* | *30* |
| *Susp. IBD*** | *67* | *-* | *103* | *-* | *61%* | *55%* | *40* | *36* |

Supplementary Table 3. Comparison between the adult participants of the current study, which comprised the subset of participants from the larger IBSEN III study with fecal samples, with all adult participants of IBSEN III at the time of inclusion to the current study. Healthy controls not included (see Table 1). *non-IBD symptomatic controls; **suspected IBD

| At inclusion → | CD | UC | IBD-U | Susp. IBD colon | Susp. IBD small intestine | Not IBD |
| --- | --- | --- | --- | --- | --- | --- |
| At one year ↓ |  |  |  |  |  |  |
| CD | 285 | 7 | 3 | 7 | 10 | - |
| UC | 5 | 606 | 6 | 3 | - | - |
| IBD-U | 2 | 2 | 21 | - | - | - |
| Susp. IBD colon | - | 2 | 2 | 11 | 1 | 1 |
| Susp. IBD small intestine | 5 | - | - | - | 21 | - |
| Not IBD | 10 | 3 | 1 | 2 | 2 | 2 |
| Missed 1-yr follow-up | 17 | 38 | 3 | 3 | 7 | 141 |

Supplementary Table 4. Summary of the number of adult participants that changed diagnosis from inclusion to 1-year follow-up.

| Sampling delay | Shannon r | Shannon p | Observed r | Observed p |
| --- | --- | --- | --- | --- |
| UC | 0.0847466 | 0.02537 | 0.05648634 | 0.1366 |
| CD | 0.02086579 | 0.6801 | 0.05856756 | 0.2467 |
| IBD-U | 0.1451774 | 0.3357 | 0.1292606 | 0.3919 |
| All | 0.04608389 | 0.08333 | 0.046191 | 0.08262 |

Supplementary Table 5. Summary of correlation between sampling delay (the number of days from diagnosis to fecal sample delivery) and alpha diversity as measured by the Shannon diversity index and the observed number of genera. R denotes correlation coefficient and p denotes p-value.

| **Variable** | **Including those with antibiotics exposure**  Adjusting for: Sex, age, BMI, sampling delay, antibiotics exposure [coefficient] | **Excluding those with antibiotics exposure**  Adjusting for: Sex, age, BMI, sampling delay [coefficient] |
| --- | --- | --- |
| Crohn’s disease vs symptomatic controls | p < 0.001 [-1.31] | p < 0.01 [-1.14] |
| Ulcerative colitis vs symptomatic controls | p < 0.001 [-1.06] | p < 0.01 [-1.03] |
| Crohn’s disease vs healthy controls | p < 0.01 [-2.00] | p < 0.05 [-1.54] |
| Ulcerative colitis vs healthy controls | p < 0.001 [-1.82] | p < 0.01 [-1.64] |
| Severe vs indolent disease course - Crohn’s disease | p < 0.001 [-1.03] | p < 0.05 [-0.87] |
| Severe vs indolent disease course - Ulcerative colitis | p < 0.001 [-1.16] | p < 0.001 [-1.18] |
| Severe vs indolent disease course - Ulcerative colitis (only mild cases) | p < 0.01 [-0.99] | p < 0.01 [-1.13] |
| Severe vs indolent disease course - Ulcerative colitis (only E2 and E3) | p < 0.01 [-1.06] | p < 0.01 [-1.03] |

Supplementary Table 6. Alpha diversity p-values and effect sizes of the generalized linear model before and after excluding samples with antibiotics exposure and adjusting for covariates.

| **Variables**  p-values from *betadisper* to test only for increased compositional variance. Normalized effect sizes given with Cohen’s f | **Including those with antibiotics exposure** | **Excluding those with antibiotics exposure** |
| --- | --- | --- |
| Crohn’s disease vs symptomatic controls | 0.18*, p* < 0.0001 | 0.15*, p* < 0.0001 |
| Ulcerative colitis vs symptomatic controls | 0.12*, p* < 0.0001 | 0.12*, p* < 0.0001 |
| IBD-U vs symptomatic controls | 0.05, *p* = 0.03 | 0.10, *p* = 0.006 |
| Crohn’s disease vs healthy controls | 0.33*, p* < 0.0001 | 0.30*, p* < 0.0001 |
| Ulcerative colitis vs healthy controls | 0.24*, p* < 0.0001 | 0.24*, p* < 0.0001 |
| IBD-U vs healthy controls | 0.34*, p* < 0.0001 | 0.36*, p* < 0.0001 |

Supplementary Table 7. Results from *betadisp* showing differences in amount of variance (dispersal) when comparing different disease groups, before and after excluding samples exposed to antibiotics.

| **Population** | **Groups** | **Gained** | **Lost** | **Reference** |
| --- | --- | --- | --- | --- |
| No antibiotics | Symptomatic non-IBD versus CD |  | *Hungatella, Lachnospiraceae ND3007 group* | Figure 2B |
| No antibiotics | Symptomatic non-IBD versus UC |  | *Holdemania, Bilophila* | Figure 2B |
| No antibiotics | CD versus UC | *Lachnospiraceae family* | *Collinsella, Blautia* | Figure 2B |
| No antibiotics | CD severe |  |  | Figure 5B |
| No antibiotics | UC severe |  | *Oscillospiraceae family, Actinomyces, Odoribacter* | Figure 5B |

Supplementary Table 8. Differential abundance analysis of the microbiome profiles of those not exposed to antibiotics compared to the results from the total population (*q* < 0.05).

| Cohort/Continent | Youden’s index | PPV | NPV | Sensitivity | Specificity | AUC |
| --- | --- | --- | --- | --- | --- | --- |
| IBSEN III | 0.79 | 0.46 | 0.86 | 0.52 | 0.83 | 0.72 |
| IBSEN III* | 0.79 | 0.23 | 0.94 | 0.57 | 0.77 | 0.68 |
| Asia | 0.34 | 0.74 | 0.62 | 0.55 | 0.79 | 0.69 |
| Europe | 0.39 | 0.47 | 0.86 | 0.68 | 0.71 | 0.73 |
| North America | 0.19 | 0.41 | 0.77 | 0.47 | 0.73 | 0.59 |

Supplementary Table 9. Details on the performance of the UC-CD index on classifying the study- and validation populations. *Excluding UC E1 (proctitis) and Crohn’s with ileal involvement (i.e. E2/3 UC vs colonic Crohn’s).

*[Supplementary Table 10 is attached in a separate pdf]*

Supplementary Table 10. Details on the performance of machine learning models both including and excluding samples with antibiotics exposure, given differing combinations of data types (microbiome, biochemical or clinical) and variables, and tested on IBSEN III-data not used in model training (i.e. the testing-fraction of the train/test-split).

| *Cutoff 0.5 for predicting CD vs UC*  *(Cutoff 0.66)* | *Sensitivity* | *Specificity* | *Positive Prediction* | *Negative Prediction* | *AUC* |
| --- | --- | --- | --- | --- | --- |
| Asia | 0.98 (0.86) | 0.18 (0.40) | 0.53 (0.58) | 0.90 (0.76) | 0.70 |
| Europe | 0.92 (0.76) | 0.36 (0.63) | 0.79 (0.84) | 0.65 (0.50) | 0.76 |
| North America | 0.90 (0.73) | 0.24 (0.46) | 0.74 (0.77) | 0.50 (0.41) | 0.64 |

Supplementary Table 11. Summary of machine learning models trained on IBSEN III classifying IBD patients in the global validation cohort into UC or CD.
