# Supplementary material for: Fecal Microbiome Reflects Disease State and Prognosis in Inflammatory Bowel Disease in an Adult Population-Based Inception Cohort
Source: Inflamm Bowel Dis. 2025 Apr 25;31(8):2066–80. doi: 10.1093/ibd/izaf060 (PMC12491950; doi:10.1093/ibd/izaf060)
Supplement: izaf060_suppl_Supplementary_Figures_1-9 [file izaf060_suppl_supplementary_figures_1-9.pdf]

Supp. Figure 1

A)

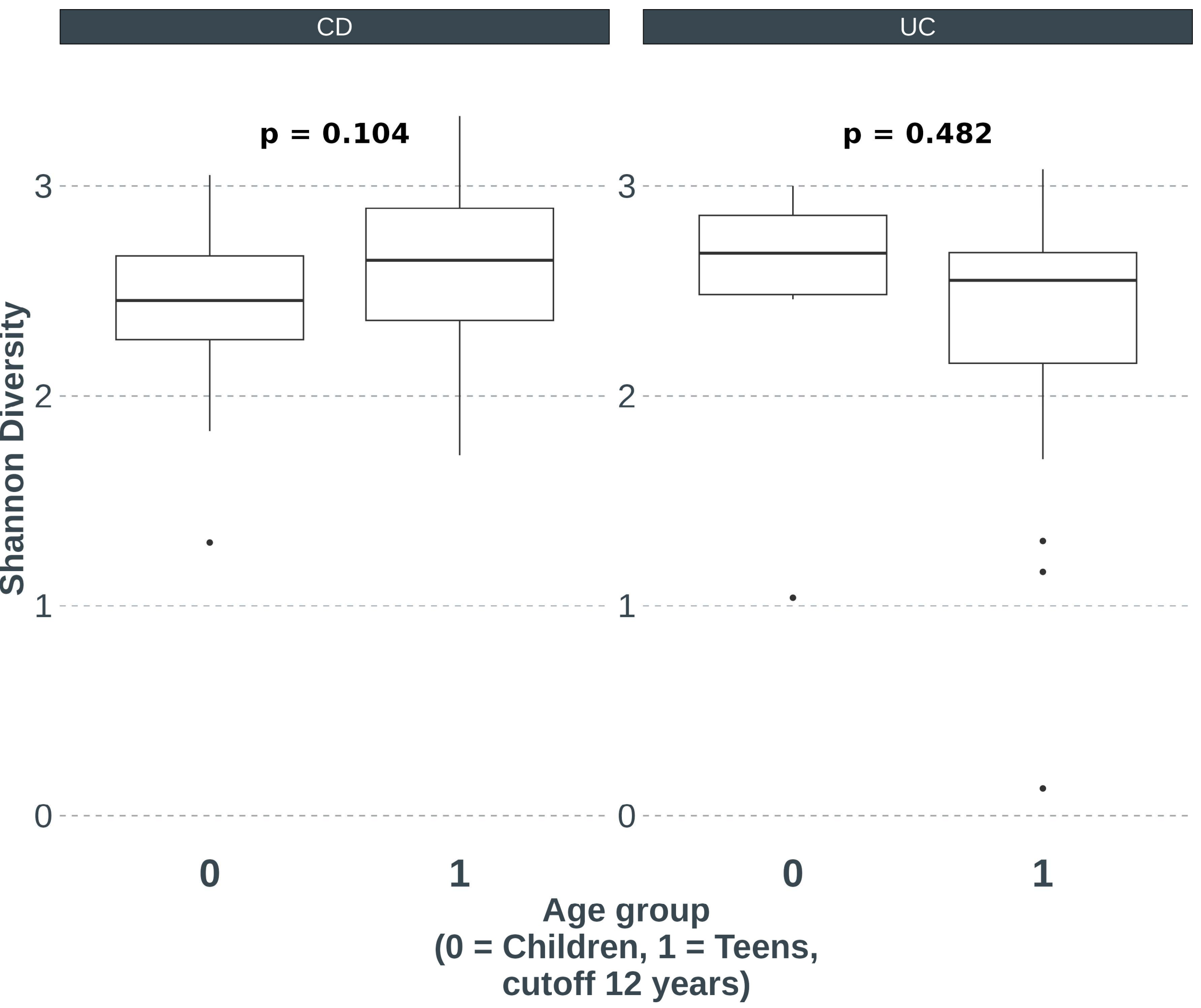

B)

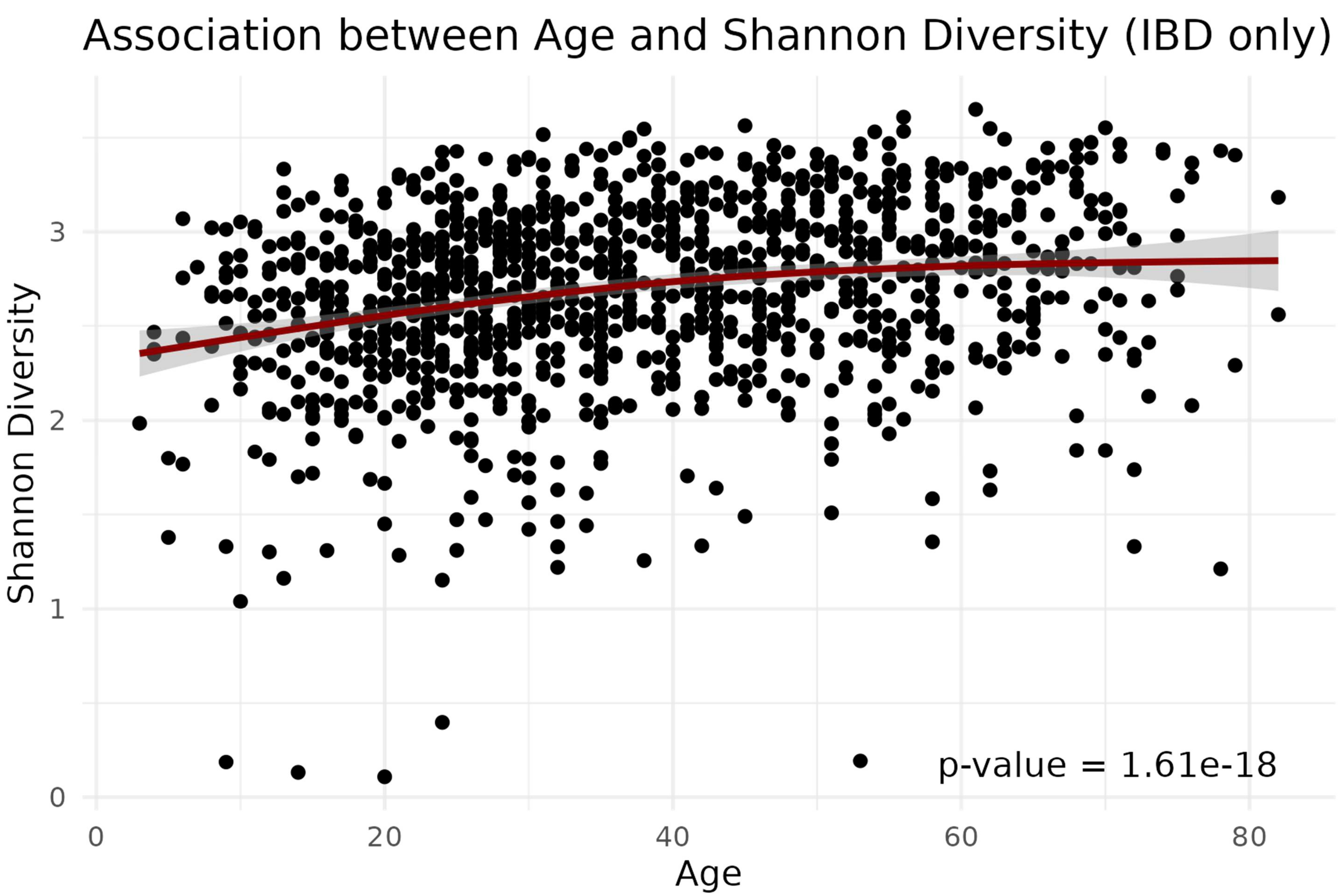

Supp. Figure 1: A) Boxplots comparing alpha diversity (Shannon diversity index) between children 12 years old or younger (age group 0) and teens aged 13 to 18 (age group 1), stratified by diagnosis (IBD only). P-values are derived from non-parametric Mann-Whitney tests.
B) Scatterplot showing the overall lower diversity among younger participants (IBD only). P value from Pearson’s correlation is reported.

Supp. Figure 2

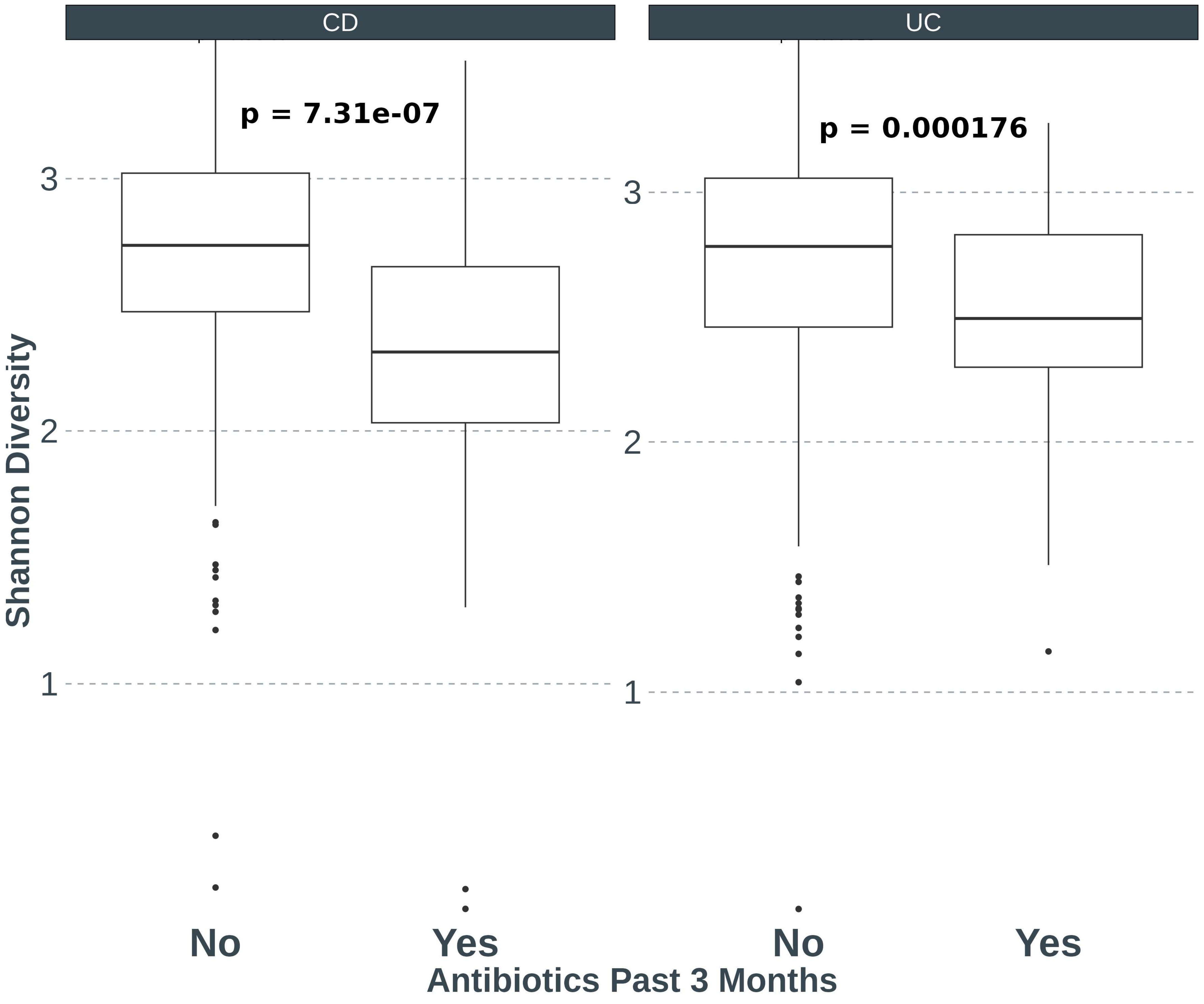

Supp. Figure 2: Boxplots showing that antibiotics use within 3 months prior to study inclusion significantly reduces microbial diversity as measured by the Shannon diversity index, both in UC and CD. P-values are derived from Mann-Whitney tests.

Supp. Figure 3

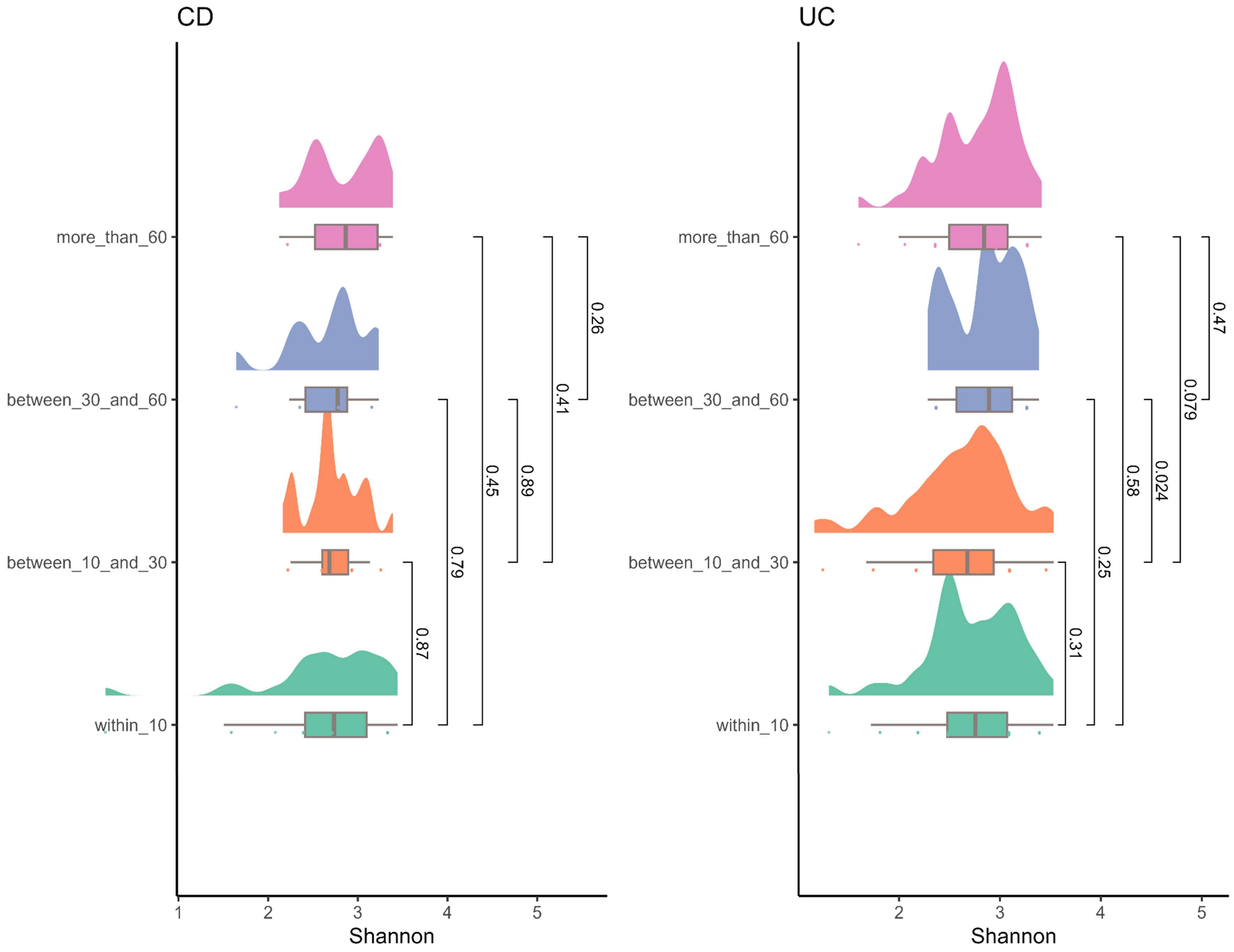

Supp. Figure 3: Boxplots and half-violin demonstrating the association between sampling delay (number of days from participant inclusion to delivered fecal sample) and Shannon diversity. The only significant comparison is between those with 10-30 days delay and those with 30-60 days delay, when subset to participants with UC. For participants with CD, no such statistical association was identified. P-values are derived from Mann-Whitney tests.

Supp. Figure 4

A)

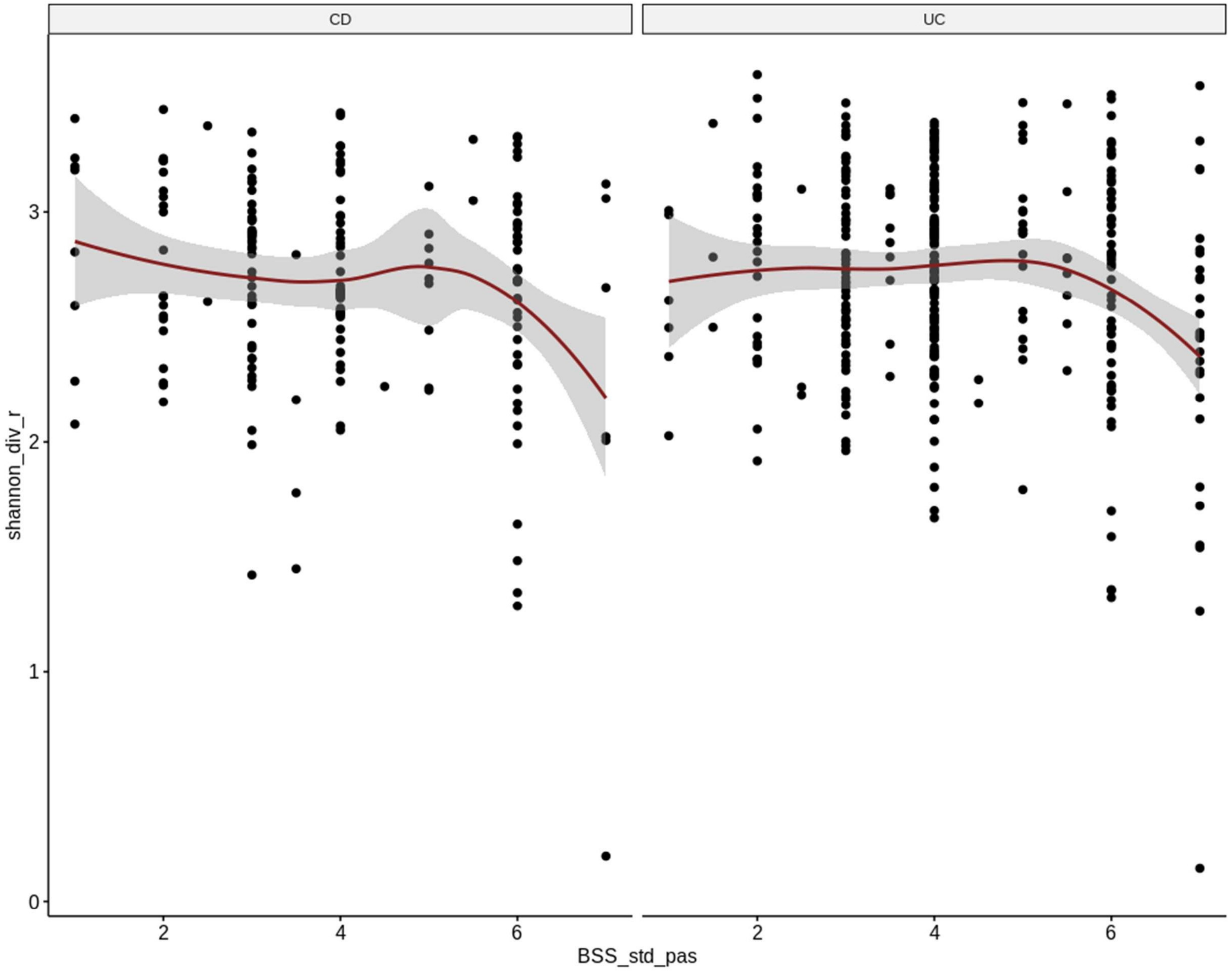

B)

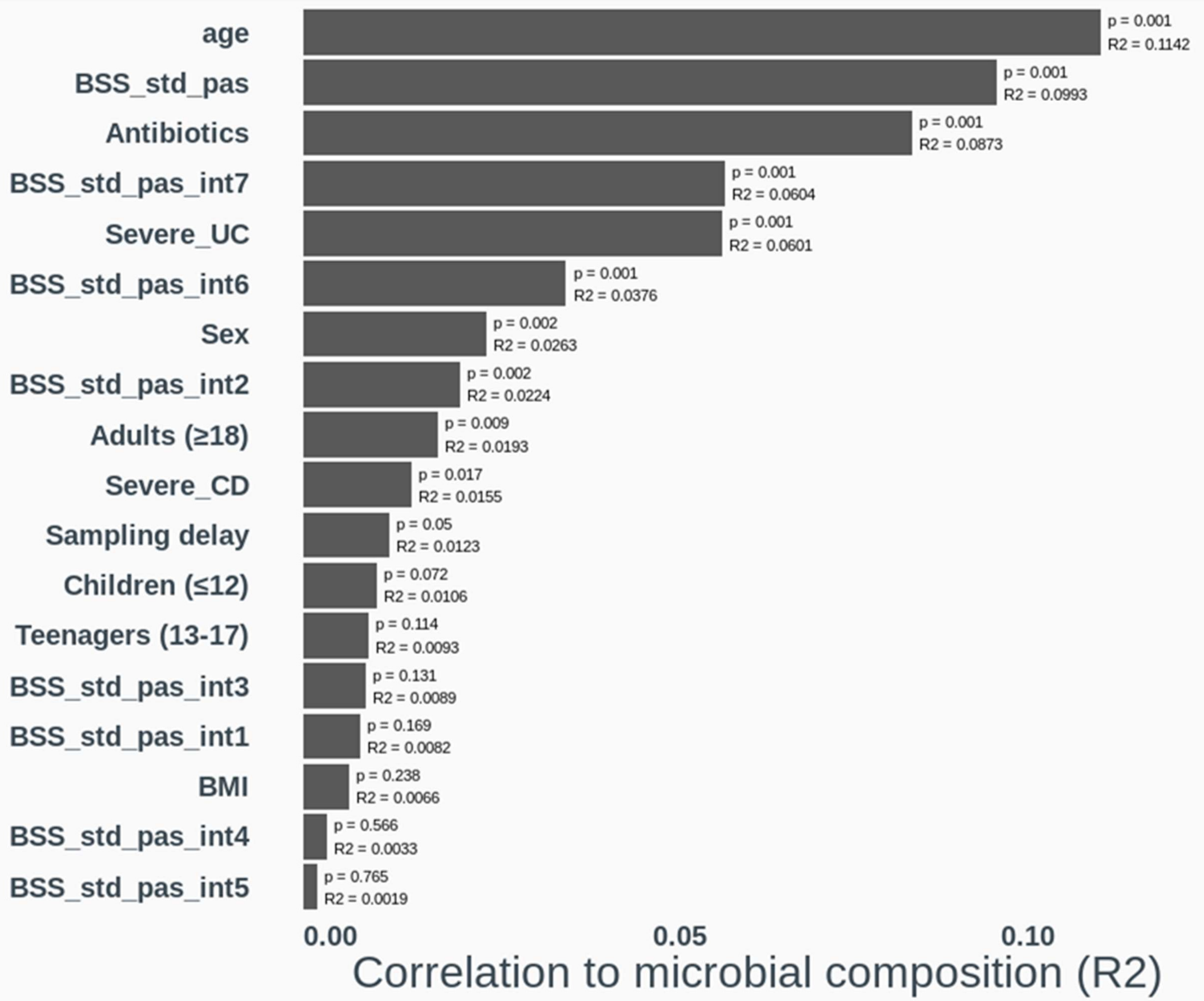

Supp. Figure 4: A) Association between participant-reported Bristol Stool Scale values (BSS_std_pas) and microbial diversity as measured by the Shannon diversity index. Only those with liquid feces (BSS_std_pas = 7) showed a reduction in diversity, but this association was not significant. B) Bristol Stool Scale values were associated with microbial composition, as tested with the envfit-function of the vegan-package in R, both when treated as a continuous variable and when converted to 7 separate dummy variables. Liquid feces (BSS_std_pas_int7) had the largest effect on composition ($p = 0.001$, $R2 = 0.06$), but also values 6 and 2 were associated with bacterial composition.

Supp. Figure 5

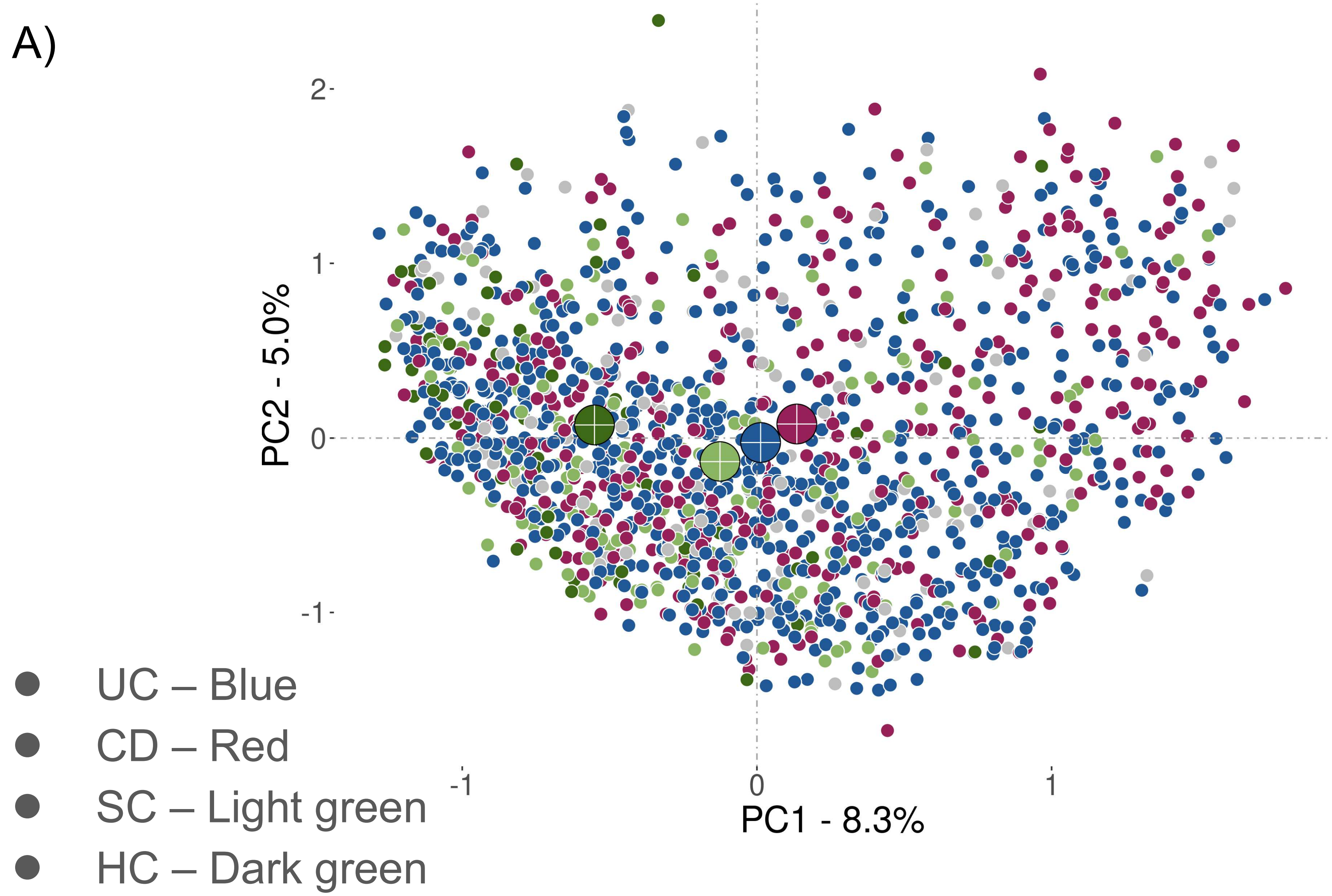

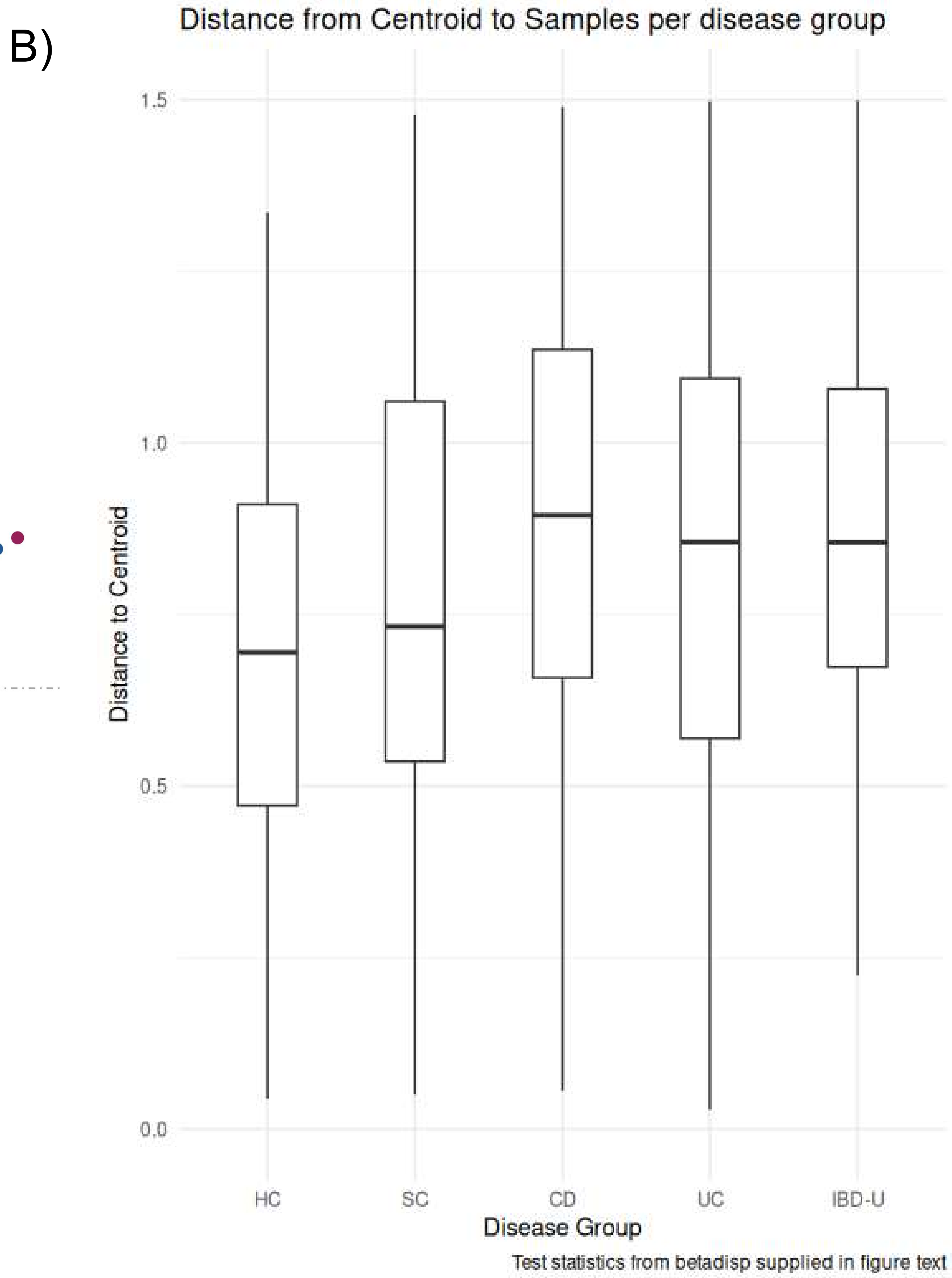

Supp. Figure 5: A) Beta diversity plot (PCoA) with distances estimated by Bray-principal dissimilarity showing individuals and centroids per disease category. The PC1 and PC2 axes denote the amount of variability explained by the principal coordinates. Ulcerative colitis (UC) is shown in blue, Crohn’s disease (CD) in red, symptomatic controls (SC) in light green, healthy controls (HC) in dark green, and participants with suspected IBD in grey. B) Boxplots showing the distances from centroids to samples within each disease group. *Betadisp* was used to test for differences in variance between pairs of IBD subtypes and control groups. HC vs UC/CD/IBD-U were all significantly different ($p<0.0001$), with HC having a smaller variance. The same was true for SC vs UC/CD ($p<0.0001$) and SC vs IBD-U ($p=0.03$). See Supplementary Table 7 for additional statistics. HC = Healthy controls; SC = non-IBD symptomatic controls; CD = Crohn's disease; UC = ulcerative colitis; IBD-U = IBD unclassified..

Supp. Figure 6

A)

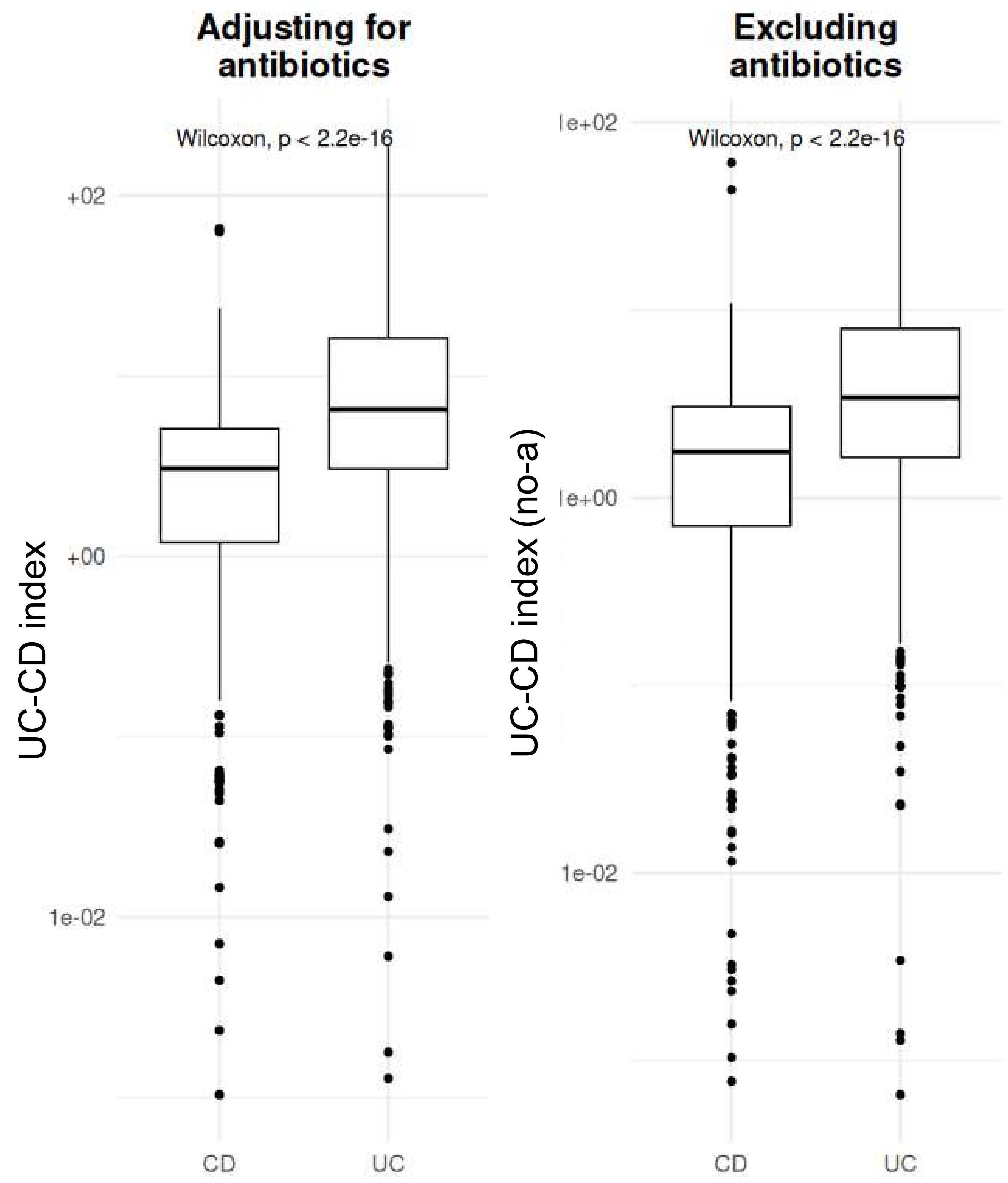

B)

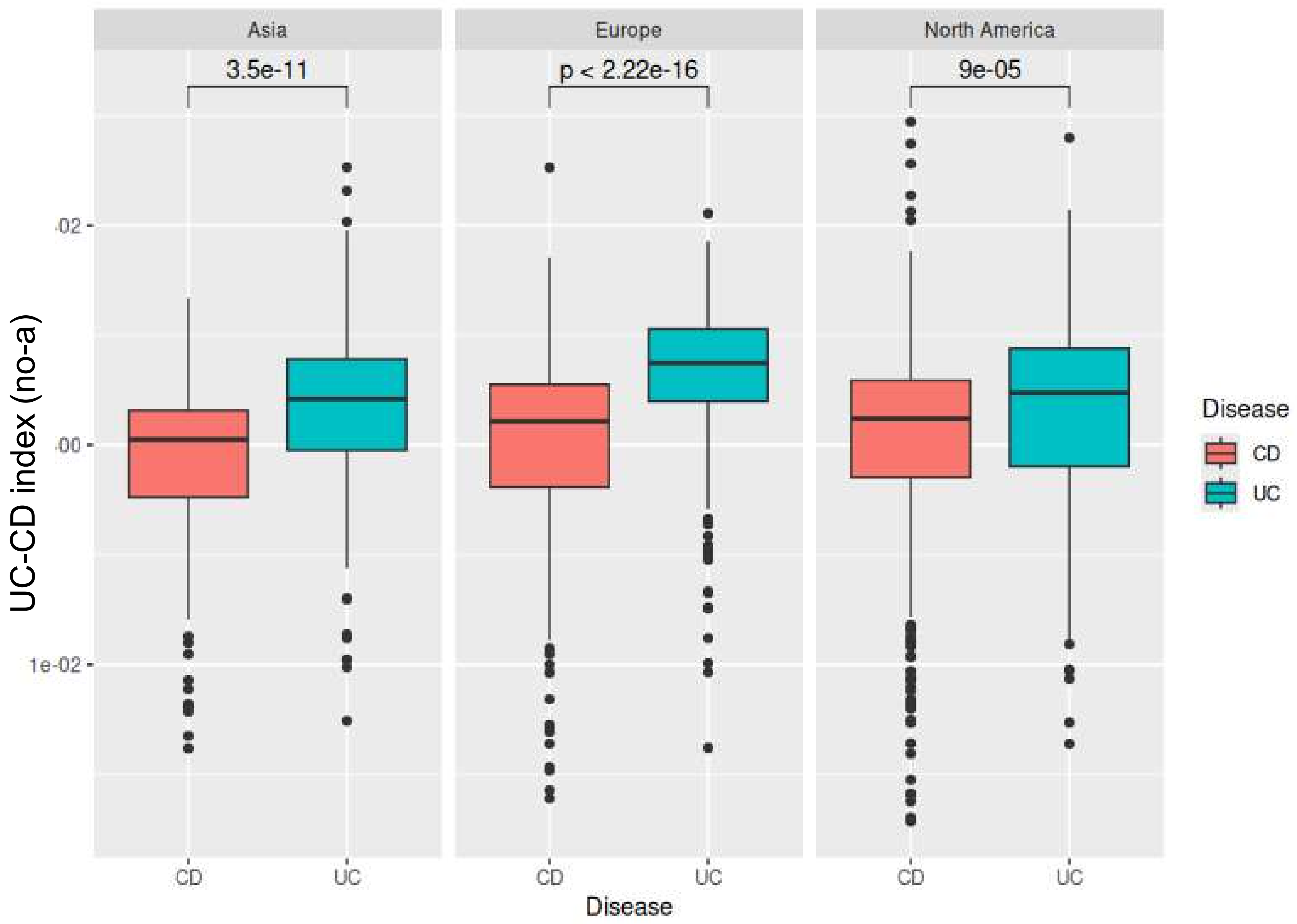

Supp. Figure 6: A) Boxplots showing UC-CD index values in the IBSEN III cohort based on differential abundance testing either adjusting for or excluding samples with antibiotics exposure (denoted as UC-CD index no-a). B) Index values based on excluding those with antibiotics separate CD and UC in the global validation cohort. For the validation plot when adjusting for antibiotics instead, see the main figure in the article (Figure 3).

Supp. Figure 7

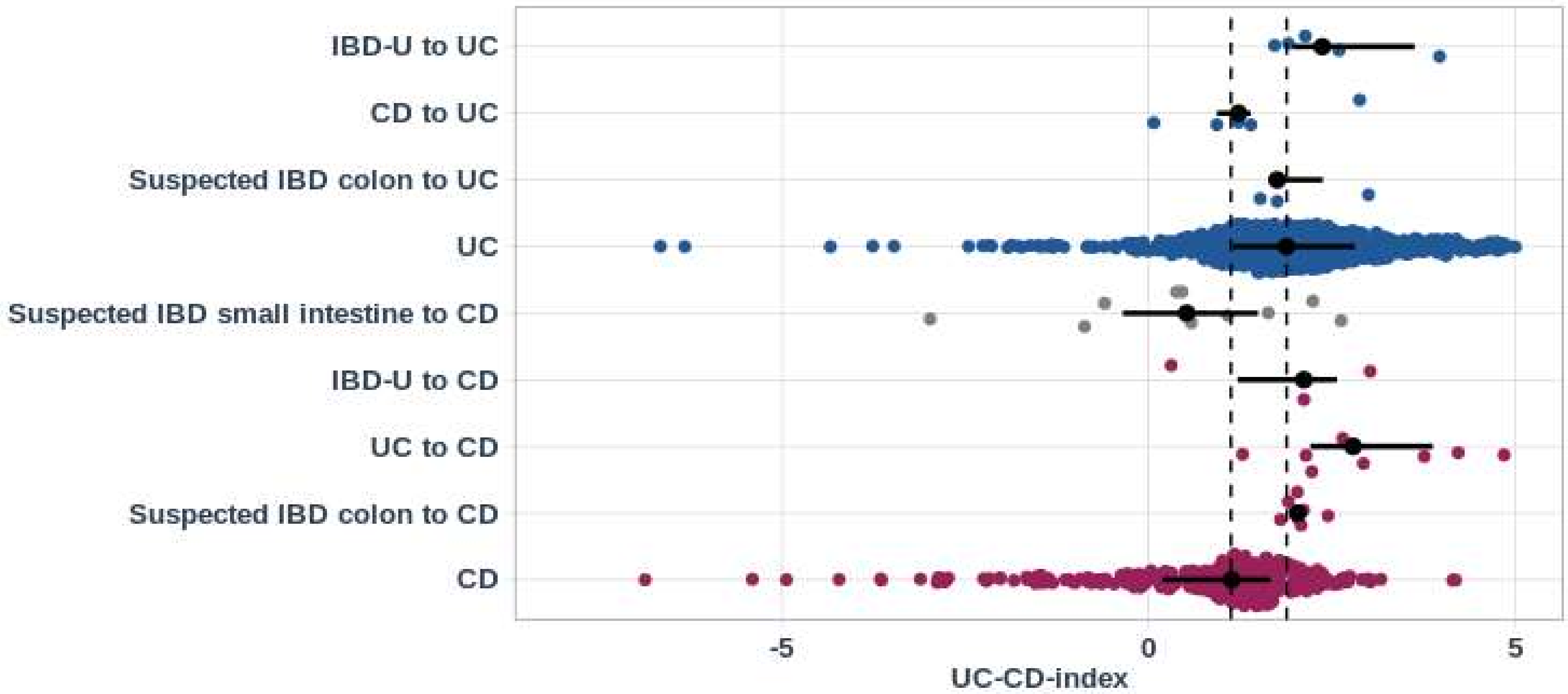

Supp. Figure 7: Summary of UC-CD index values with a focus on participants that changed diagnosis from baseline to 1-year follow-up. Those that changed from UC to CD, or vice-versa, had UC-CD index values conforming most to their original diagnosis.

Supp. Figure 8

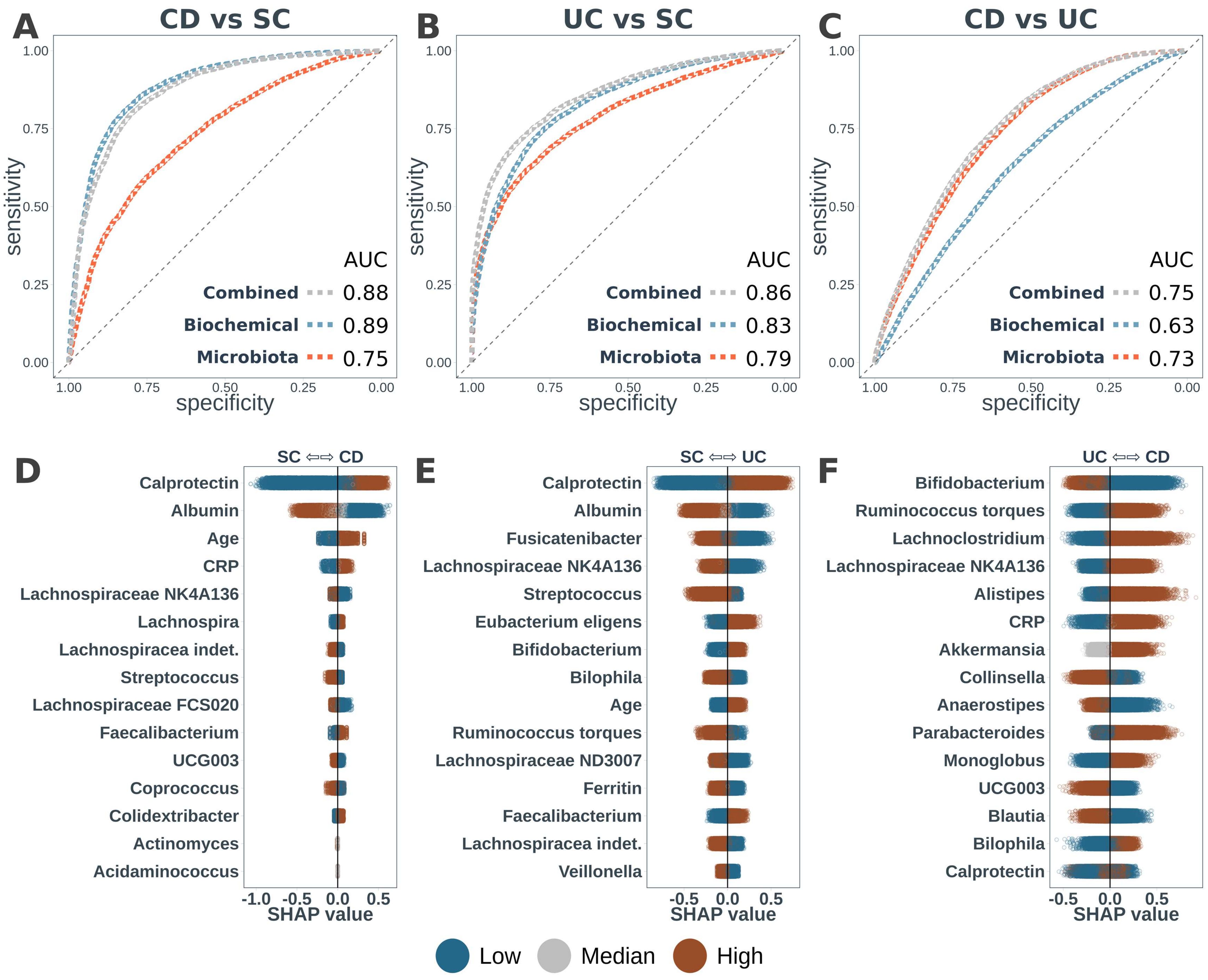

Supp. Figure 8: (A, B, C) AUC plots summarizing 100 machine learning models per plot, showing the classification ability of three different sets of variables in separating differing pairs of diagnoses. The difference between any two pairs of AUC values is at least $p < 0.001$ (Mann Whitney test). (D, E, F) SHAP summary plots visualizing the importance and direction of effect per variable. Positive SHAP values indicate a contribution towards predicting the case (i.e. UC in E, and CD in D and F), while negative SHAP values indicate a contribution towards predicting the control (i.e. SC in D and E, and UC in F). Blue colored dots represent patients with low relative values for a variable, while red colored dots represent patients with high relative values for a variable.

Supp. Figure 9

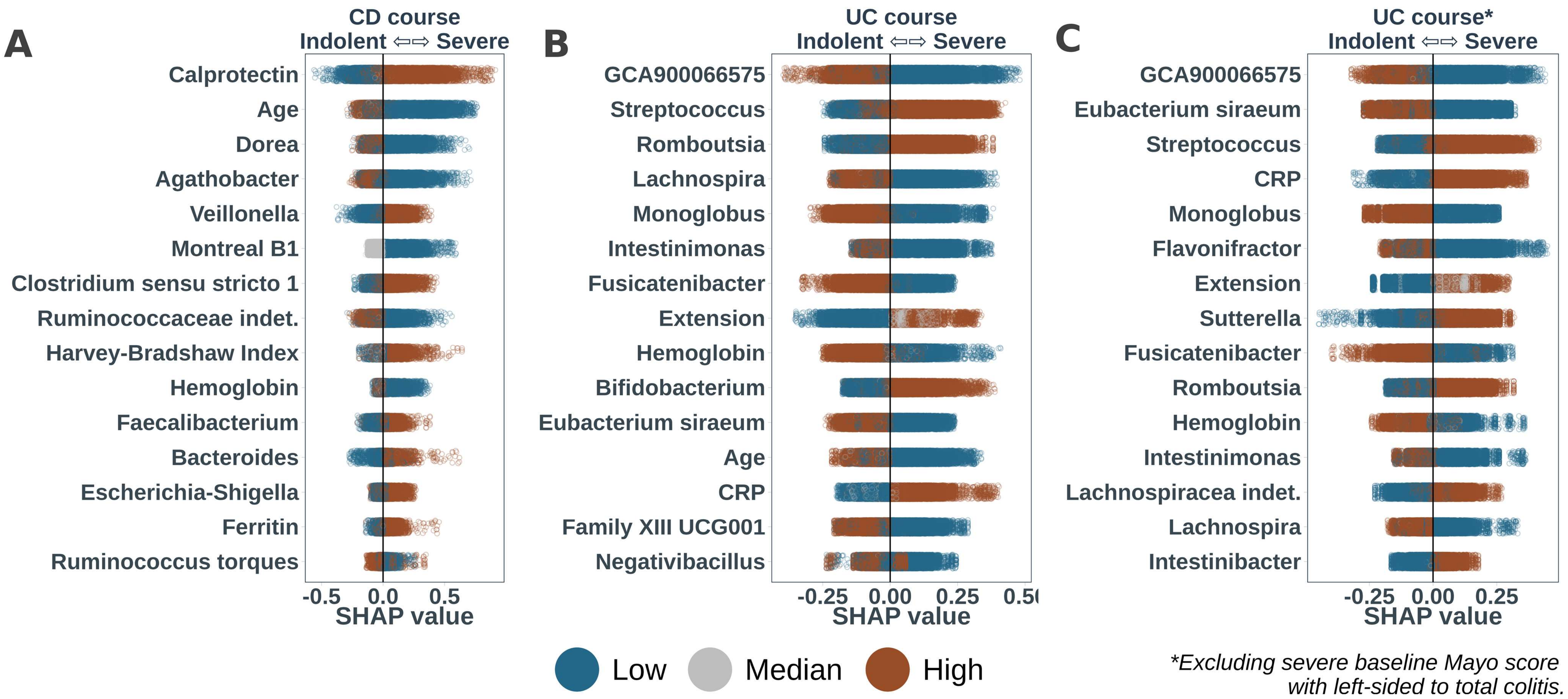

**Excluding severe baseline Mayo score with left-sided to total colitis.*

Supp. Figure 9: (A, B, C) SHAP summary plots visualizing the importance and direction of effect per variable for the classification by the GPBoost machine learning model. Positive SHAP values indicate a contribution towards predicting the case (i.e. severe disease), while negative SHAP values indicate a contribution towards predicting the control (i.e. indolent disease). Blue colored dots represent patients with low relative values for a variable, while red colored dots represent patients with high relative values for a variable.

Supp. Figure 10

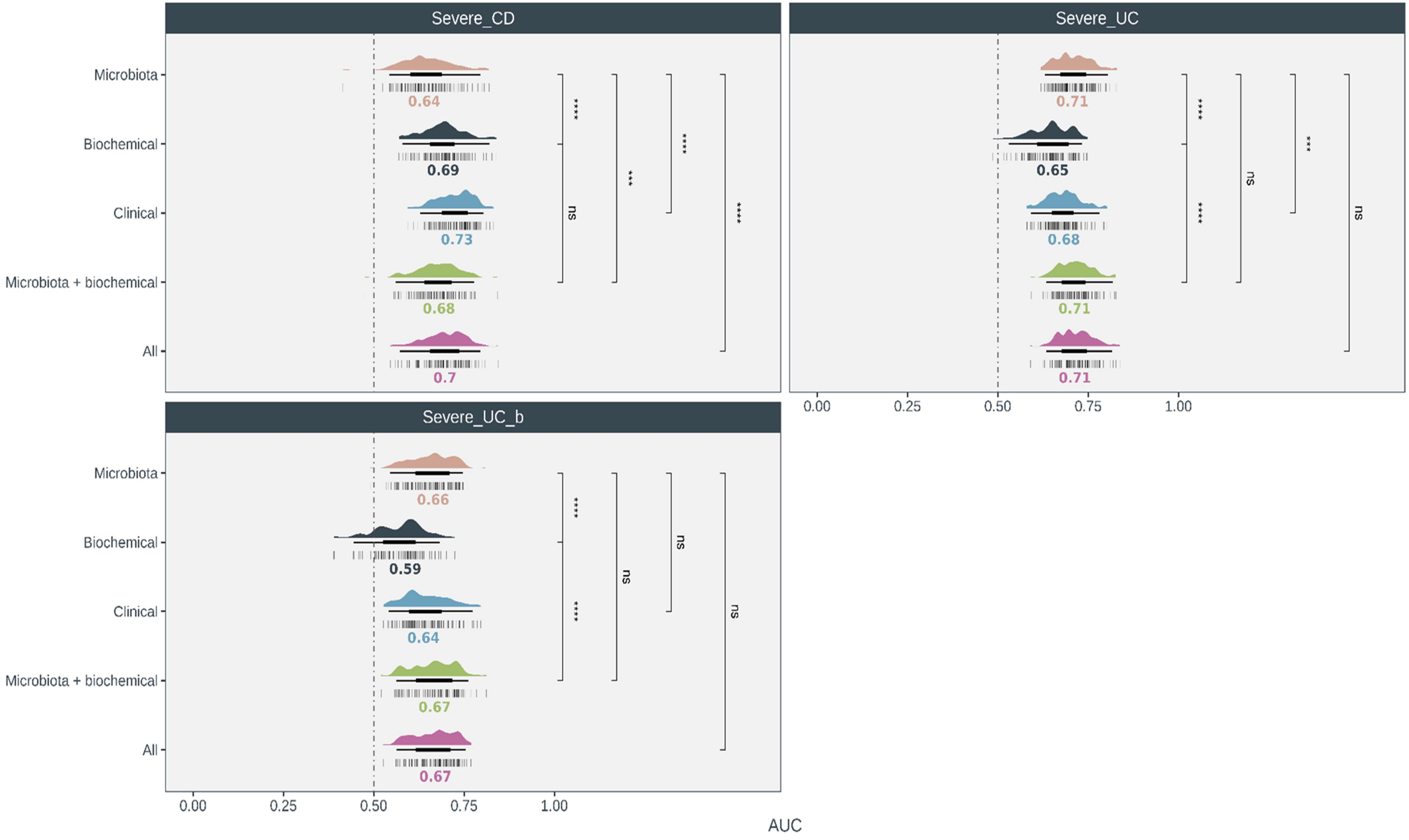

Supp. Figure 10: Summarized results from iterative machine learning runs, as described in detail in the supplementary methods. Microbiota profiles were the worst predictor of a severe disease course in CD, but outperformed both biochemical data (F-calprotectin, C-reactive protein, and others) and clinical features (e.g. Mayo endoscopic sub score) in UC. This remained the case when only looking at UC participants presenting with a mild disease at baseline ("Severe_UC_b" = Mayo endoscopic score below 3, and/or isolated proctitis). * $p<0.05$, ** $p<0.01$, *** $p<0.001$, **** $p<0.0001$, ns = $p > 0.05$, Mann-Whitney tests of AUC values from 100 runs of GPBoost per set of predictor variables.

Supp. Figure 11

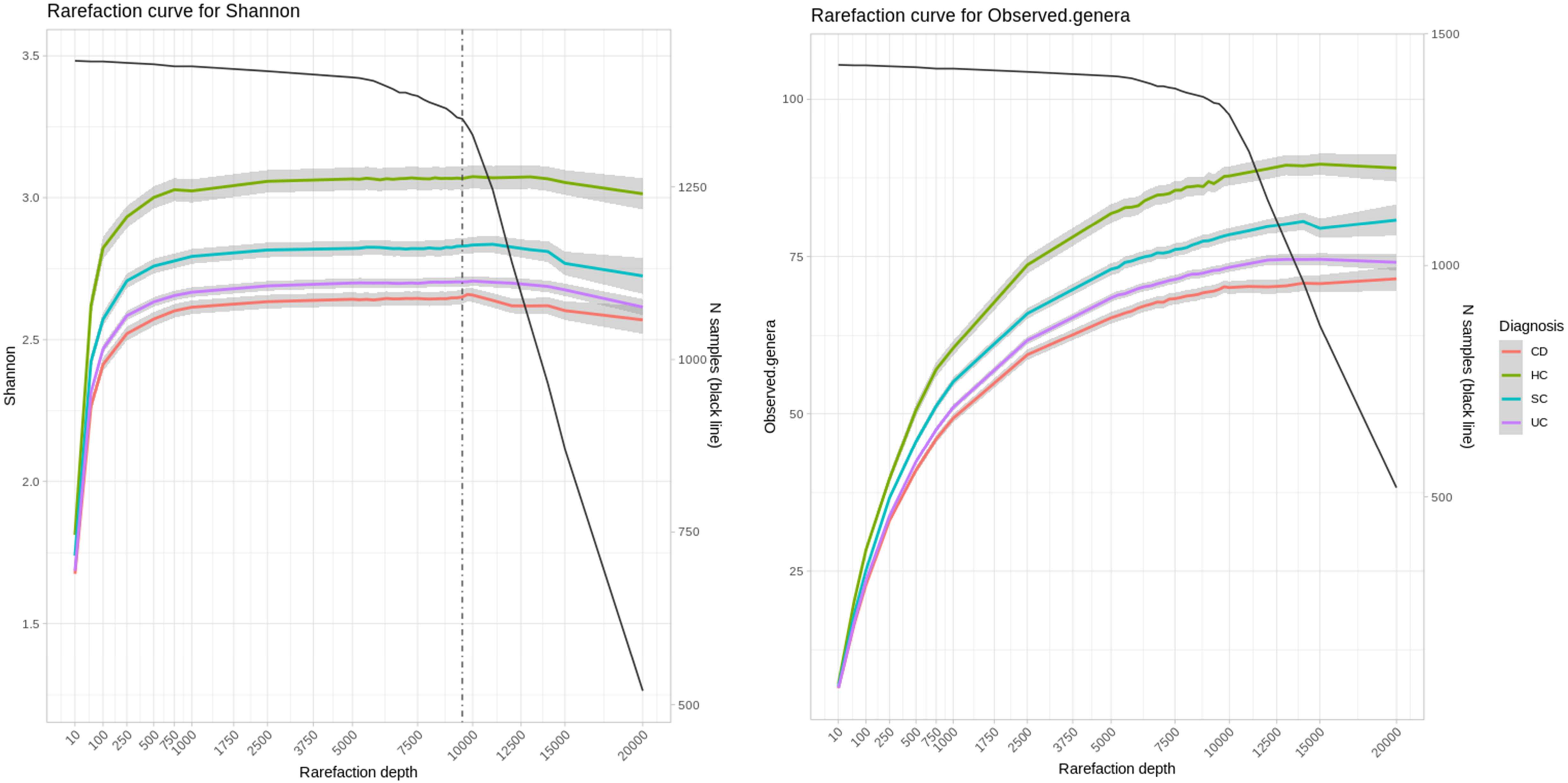

Supp. Figure 11: Rarefaction curves showing the chosen cut-off at 9500 reads, identified as a balanced point between excluding as few samples as possible while still keeping a high number of reads representative of the microbial composition and diversity.

Supp. Figure 12

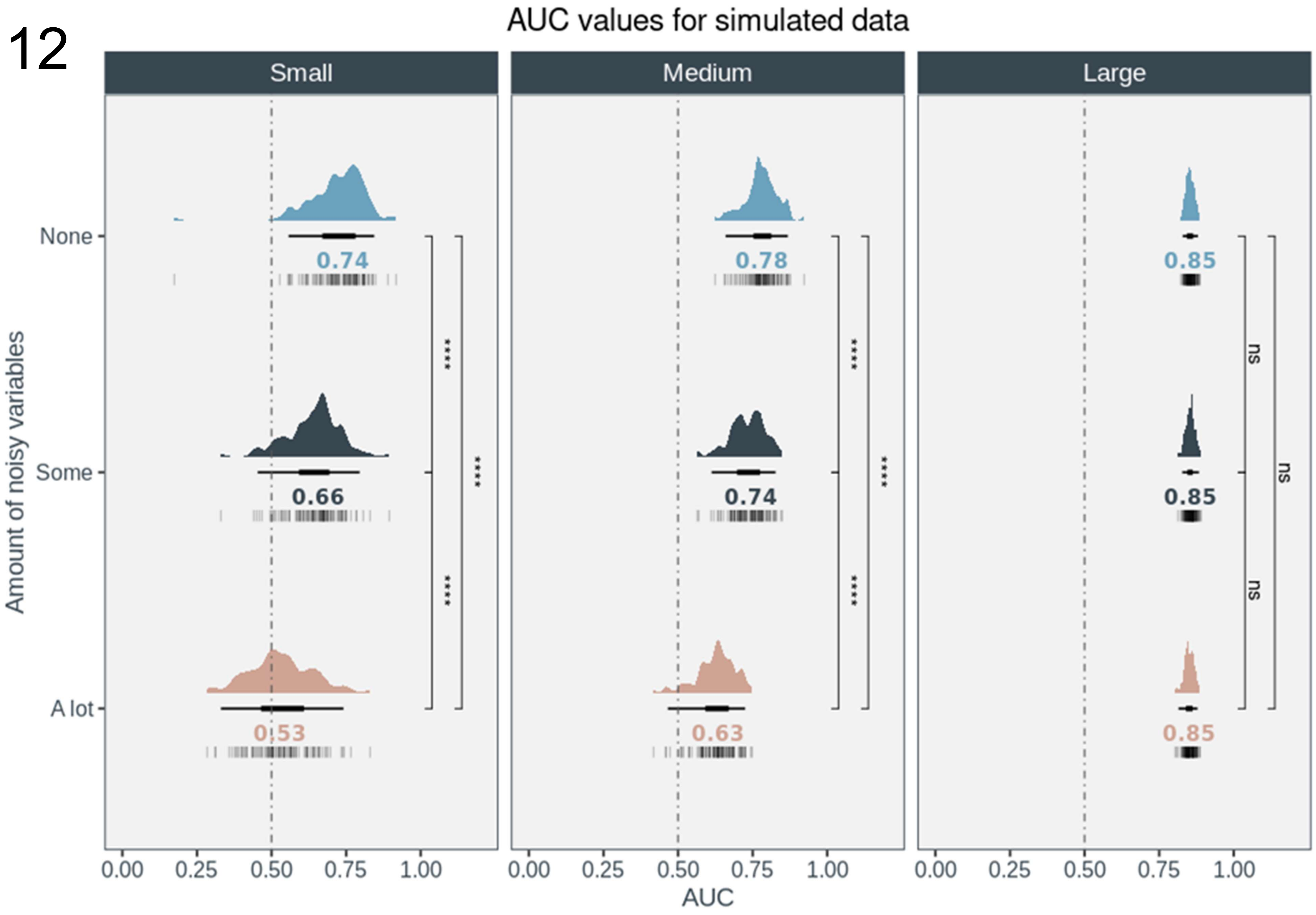

Supp. Figure 12: Summarized results from a synthetic experiment showing that machine learning, here represented by XGBoost, can filter out noisy variables only when the data set is sufficiently large. When a large amount of noisy variables (995 vs 5 “real” variables) are introduced in a small dataset (20 cases and 100 controls), AUC-values had a median of 0.53 and were on average not significantly better than a random model (interquartile range contains 0.5). This experiment was partly constructed and run to explain why some of the machine learning models performed worse after bacterial data were introduced to clinical features (see Supp. Fig 6). Sizes (cases/controls): Small (20/100), Medium (50/200), Large (500/500). Noise (n real variables/n noisy variables): None (5/0), Some (5/95), A lot (5/995)
