# Supplementary material for: Fecal Microbiome Reflects Disease State and Prognosis in Inflammatory Bowel Disease in an Adult Population-Based Inception Cohort
Source: Inflamm Bowel Dis. 2025 Apr 25;31(8):2066–80. doi: 10.1093/ibd/izaf060 (PMC12491950; doi:10.1093/ibd/izaf060)
Supplement: izaf060_suppl_Supplementary_Tables_1 [file izaf060_suppl_supplementary_tables_1.pdf]

	Asia		Europe		North America		Overall	
	CD (N=191)	UC (N=202)	CD (N=583)	UC (N=222)	CD (N=568)	UC (N=232)	CD (N=1342)	UC (N=656)
**Host_Age**								
Mean (SD)	31.4 (8.58)	13.7 (3.40)	35.9 (13.9)	42.2 (14.3)	25.8 (18.0)	34.2 (19.6)	29.7 (17.2)	34.9 (18.7)
Median [Min, Max]	32.0 [22.0, 45.0]	12.5 [10.0, 22.0]	35.0 [8.29, 76.0]	42.0 [5.00, 75.0]	16.2 [3.83, 76.0]	33.0 [6.75, 80.0]	28.0 [3.83, 76.0]	35.0 [5.00, 80.0]
Missing	184 (96.3%)	186 (92.1%)	322 (55.2%)	162 (73.0%)	149 (26.2%)	110 (47.4%)	655 (48.8%)	458 (69.8%)
**host_body_mass_index**								
Mean (SD)	NA (NA)	NA (NA)	23.4 (5.16)	26.2 (18.9)	22.3 (4.12)	23.8 (5.13)	23.0 (4.84)	24.8 (13.1)
Median [Min, Max]	NA [NA, NA]	NA [NA, NA]	22.5 [13.0, 40.6]	24.2 [14.9, 149]	21.0 [15.2, 33.5]	23.1 [14.0, 45.7]	22.1 [13.0, 40.6]	23.4 [14.0, 149]
Missing	191 (100%)	202 (100%)	510 (87.5%)	175 (78.8%)	529 (93.1%)	173 (74.6%)	1230 (91.7%)	550 (83.8%)
**host_sex**								
female	27 (14.1%)	10 (5.0%)	195 (33.4%)	62 (27.9%)	259 (45.6%)	102 (44.0%)	481 (35.8%)	174 (26.5%)
male	42 (22.0%)	7 (3.5%)	137 (23.5%)	60 (27.0%)	262 (46.1%)	87 (37.5%)	441 (32.9%)	154 (23.5%)
Missing	122 (63.9%)	185 (91.6%)	251 (43.1%)	100 (45.0%)	47 (8.3%)	43 (18.5%)	420 (31.3%)	328 (50.0%)
**BioProject**								
PRJNA418765	7 (3.7%)	0 (0%)	145 (24.9%)	0 (0%)	145 (25.5%)	0 (0%)	297 (22.1%)	0 (0%)
PRJNA422193	70 (36.6%)	62 (30.7%)	0 (0%)	0 (0%)	0 (0%)	0 (0%)	70 (5.2%)	62 (9.5%)
PRJNA428898	9 (4.7%)	0 (0%)	0 (0%)	0 (0%)	0 (0%)	0 (0%)	9 (0.7%)	0 (0%)
PRJNA450540	62 (32.5%)	0 (0%)	0 (0%)	0 (0%)	0 (0%)	0 (0%)	62 (4.6%)	0 (0%)
PRJNA606913	27 (14.1%)	0 (0%)	0 (0%)	0 (0%)	0 (0%)	0 (0%)	27 (2.0%)	0 (0%)
PRJNA684584	16 (8.4%)	41 (20.3%)	0 (0%)	0 (0%)	0 (0%)	0 (0%)	16 (1.2%)	41 (6.3%)
PRJDB4871	0 (0%)	16 (7.9%)	0 (0%)	0 (0%)	0 (0%)	0 (0%)	0 (0%)	16 (2.4%)
PRJEB11419	0 (0%)	1 (0.5%)	26 (4.5%)	31 (14.0%)	40 (7.0%)	60 (25.9%)	66 (4.9%)	92 (14.0%)
PRJNA596333	0 (0%)	58 (28.7%)	0 (0%)	0 (0%)	0 (0%)	0 (0%)	0 (0%)	58 (8.8%)
PRJNA681685	0 (0%)	24 (11.9%)	0 (0%)	0 (0%)	0 (0%)	0 (0%)	0 (0%)	24 (3.7%)
PRJEB11845	0 (0%)	0 (0%)	66 (11.3%)	0 (0%)	0 (0%)	0 (0%)	66 (4.9%)	0 (0%)
PRJEB14084	0 (0%)	0 (0%)	26 (4.5%)	0 (0%)	0 (0%)	0 (0%)	26 (1.9%)	0 (0%)
PRJEB18780	0 (0%)	0 (0%)	16 (2.7%)	0 (0%)	0 (0%)	0 (0%)	16 (1.2%)	0 (0%)
PRJEB20520	0 (0%)	0 (0%)	63 (10.8%)	0 (0%)	0 (0%)	0 (0%)	63 (4.7%)	0 (0%)
PRJEB21819	0 (0%)	0 (0%)	7 (1.2%)	0 (0%)	0 (0%)	0 (0%)	7 (0.5%)	0 (0%)
PRJEB3206	0 (0%)	0 (0%)	10 (1.7%)	0 (0%)	0 (0%)	0 (0%)	10 (0.7%)	0 (0%)
PRJEB33031	0 (0%)	0 (0%)	16 (2.7%)	0 (0%)	0 (0%)	0 (0%)	16 (1.2%)	0 (0%)
PRJEB7166	0 (0%)	0 (0%)	16 (2.7%)	0 (0%)	0 (0%)	0 (0%)	16 (1.2%)	0 (0%)
PRJEB7772	0 (0%)	0 (0%)	17 (2.9%)	31 (14.0%)	26 (4.6%)	36 (15.5%)	43 (3.2%)	67 (10.2%)
PRJNA391149	0 (0%)	0 (0%)	3 (0.5%)	11 (5.0%)	0 (0%)	0 (0%)	3 (0.2%)	11 (1.7%)
PRJNA414072	0 (0%)	0 (0%)	60 (10.3%)	43 (19.4%)	72 (12.7%)	61 (26.3%)	132 (9.8%)	104 (15.9%)
PRJNA514452	0 (0%)	0 (0%)	50 (8.6%)	0 (0%)	0 (0%)	0 (0%)	50 (3.7%)	0 (0%)
PRJNA565903	0 (0%)	0 (0%)	14 (2.4%)	0 (0%)	0 (0%)	0 (0%)	14 (1.0%)	0 (0%)
PRJNA603658	0 (0%)	0 (0%)	15 (2.6%)	0 (0%)	0 (0%)	0 (0%)	15 (1.1%)	0 (0%)
PRJNA757573	0 (0%)	0 (0%)	33 (5.7%)	16 (7.2%)	0 (0%)	0 (0%)	33 (2.5%)	16 (2.4%)
PRJEB11841	0 (0%)	0 (0%)	0 (0%)	23 (10.4%)	0 (0%)	0 (0%)	0 (0%)	23 (3.5%)
PRJNA316059	0 (0%)	0 (0%)	0 (0%)	14 (6.3%)	0 (0%)	0 (0%)	0 (0%)	14 (2.1%)
PRJNA368966	0 (0%)	0 (0%)	0 (0%)	31 (14.0%)	0 (0%)	0 (0%)	0 (0%)	31 (4.7%)
PRJNA515212	0 (0%)	0 (0%)	0 (0%)	7 (3.2%)	0 (0%)	0 (0%)	0 (0%)	7 (1.1%)
PRJNA645883	0 (0%)	0 (0%)	0 (0%)	15 (6.8%)	0 (0%)	0 (0%)	0 (0%)	15 (2.3%)
PRJEB13680	0 (0%)	0 (0%)	0 (0%)	0 (0%)	215 (37.9%)	23 (9.9%)	215 (16.0%)	23 (3.5%)
PRJNA324147	0 (0%)	0 (0%)	0 (0%)	0 (0%)	26 (4.6%)	10 (4.3%)	26 (1.9%)	10 (1.5%)
PRJNA380944	0 (0%)	0 (0%)	0 (0%)	0 (0%)	7 (1.2%)	16 (6.9%)	7 (0.5%)	16 (2.4%)
PRJNA388210	0 (0%)	0 (0%)	0 (0%)	0 (0%)	19 (3.3%)	0 (0%)	19 (1.4%)	0 (0%)
PRJNA450340	0 (0%)	0 (0%)	0 (0%)	0 (0%)	18 (3.2%)	19 (8.2%)	18 (1.3%)	19 (2.9%)
PRJNA596546	0 (0%)	0 (0%)	0 (0%)	0 (0%)	0 (0%)	7 (3.0%)	0 (0%)	7 (1.1%)
**Instrument**								
Illumina HiSeq 2500	27 (14.1%)	0 (0%)	0 (0%)	0 (0%)	0 (0%)	0 (0%)	27 (2.0%)	0 (0%)
Illumina MiSeq	164 (85.9%)	186 (92.1%)	410 (70.3%)	196 (88.3%)	542 (95.4%)	222 (95.7%)	1116 (83.2%)	604 (92.1%)
454 GS FLX Titanium	0 (0%)	16 (7.9%)	155 (26.6%)	0 (0%)	0 (0%)	0 (0%)	155 (11.6%)	16 (2.4%)
454 GS Junior	0 (0%)	0 (0%)	3 (0.5%)	11 (5.0%)	0 (0%)	0 (0%)	3 (0.2%)	11 (1.7%)
Ion Torrent PGM	0 (0%)	0 (0%)	15 (2.6%)	15 (6.8%)	0 (0%)	0 (0%)	15 (1.1%)	15 (2.3%)
Illumina HiSeq 2000	0 (0%)	0 (0%)	0 (0%)	0 (0%)	26 (4.6%)	10 (4.3%)	26 (1.9%)	10 (1.5%)
**X16s_region**								
V3-V4	27 (14.1%)	24 (11.9%)	130 (22.3%)	97 (43.7%)	72 (12.7%)	61 (26.3%)	229 (17.1%)	182 (27.7%)
V4	155 (81.2%)	104 (51.5%)	254 (43.6%)	85 (38.3%)	496 (87.3%)	164 (70.7%)	905 (67.4%)	353 (53.8%)
V4-V5	9 (4.7%)	0 (0%)	0 (0%)	15 (6.8%)	0 (0%)	0 (0%)	9 (0.7%)	15 (2.3%)
V1-V2	0 (0%)	16 (7.9%)	26 (4.5%)	0 (0%)	0 (0%)	0 (0%)	26 (1.9%)	16 (2.4%)
V3	0 (0%)	58 (28.7%)	0 (0%)	14 (6.3%)	0 (0%)	0 (0%)	0 (0%)	72 (11.0%)
V1-V3	0 (0%)	0 (0%)	158 (27.1%)	11 (5.0%)	0 (0%)	0 (0%)	158 (11.8%)	11 (1.7%)
V2, V3, V4, V6-V7, V8, V9	0 (0%)	0 (0%)	15 (2.6%)	0 (0%)	0 (0%)	0 (0%)	15 (1.1%)	0 (0%)
V3-V6	0 (0%)	0 (0%)	0 (0%)	0 (0%)	0 (0%)	7 (3.0%)	0 (0%)	7 (1.1%)
