# Supplementary material for: Fecal Microbiome Reflects Disease State and Prognosis in Inflammatory Bowel Disease in an Adult Population-Based Inception Cohort
Source: Inflamm Bowel Dis. 2025 Apr 25;31(8):2066–80. doi: 10.1093/ibd/izaf060 (PMC12491950; doi:10.1093/ibd/izaf060)
Supplement: izaf060_suppl_Supplementary_Tables_10 [file izaf060_suppl_supplementary_tables_10.pdf]

Antibiotics	*Variable*	*Data*	*AUC*	*Sensitivity*	*Specificity*	*Positive Predictive Value*	*Negative Predictive Value*
Excluded	CD vs SC	Bacteria	0,74	0,67	0,74	0,85	0,52
Included	CD vs SC	Bacteria	0,74	0,67	0,73	0,85	0,51
Excluded	CD vs SC	Bacteria and biochemical	0,88	0,82	0,82	0,91	0,69
Included	CD vs SC	Bacteria and biochemical	0,88	0,83	0,81	0,91	0,68
Excluded	CD vs SC	Biochemical	0,89	0,84	0,84	0,92	0,72
Included	CD vs SC	Biochemical	0,89	0,83	0,84	0,92	0,70
Excluded	IBD vs SC	Bacteria	0,76	0,65	0,79	0,96	0,26
Included	IBD vs SC	Bacteria	0,76	0,66	0,77	0,95	0,26
Excluded	IBD vs SC	Bacteria and biochemical	0,87	0,78	0,84	0,97	0,36
Included	IBD vs SC	Bacteria and biochemical	0,87	0,77	0,84	0,97	0,36
Excluded	IBD vs SC	Biochemical	0,85	0,78	0,81	0,97	0,36
Included	IBD vs SC	Biochemical	0,85	0,78	0,82	0,97	0,36
Excluded	UC vs SC	Bacteria	0,78	0,65	0,82	0,95	0,34
Included	UC vs SC	Bacteria	0,79	0,67	0,82	0,95	0,36
Excluded	UC vs SC	Bacteria and biochemical	0,85	0,74	0,85	0,96	0,42
Included	UC vs SC	Bacteria and biochemical	0,86	0,75	0,86	0,96	0,43
Excluded	UC vs SC	Biochemical	0,83	0,75	0,80	0,95	0,42
Included	UC vs SC	Biochemical	0,83	0,75	0,80	0,95	0,42
Excluded	Severe CD	All	0,67	0,76	0,64	0,27	0,94
Included	Severe CD	All	0,70	0,75	0,64	0,32	0,93
Excluded	Severe CD	Bacteria	0,67	0,71	0,68	0,30	0,93
Included	Severe CD	Bacteria	0,65	0,67	0,67	0,32	0,91
Excluded	Severe CD	Bacteria and biochemical	0,67	0,72	0,68	0,29	0,94
Included	Severe CD	Bacteria and biochemical	0,68	0,73	0,65	0,31	0,92
Excluded	Severe CD	Biochemical	0,72	0,73	0,73	0,33	0,94
Included	Severe CD	Biochemical	0,69	0,69	0,71	0,34	0,92
Excluded	Severe CD	Clinical	0,72	0,78	0,68	0,31	0,95
Included	Severe CD	Clinical	0,72	0,83	0,63	0,32	0,95
Excluded	Severe UC	All	0,72	0,69	0,74	0,21	0,96
Included	Severe UC	All	0,72	0,70	0,72	0,22	0,96
Excluded	Severe UC	Bacteria	0,71	0,69	0,70	0,20	0,96
Included	Severe UC	Bacteria	0,71	0,71	0,69	0,21	0,96
Excluded	Severe UC	Bacteria and biochemical	0,72	0,70	0,73	0,21	0,96
Included	Severe UC	Bacteria and biochemical	0,71	0,69	0,72	0,22	0,96
Excluded	Severe UC	Biochemical	0,64	0,60	0,72	0,19	0,95
Included	Severe UC	Biochemical	0,65	0,62	0,70	0,19	0,95
Excluded	Severe UC	Clinical	0,68	0,71	0,65	0,17	0,96
Included	Severe UC	Clinical	0,68	0,73	0,62	0,18	0,96
Excluded	Severe UC (mild	All	0,69	0,70	0,68	0,18	0,97

	baseline)						
Included	Severe UC (mild baseline)	All	0,66	0,69	0,66	0,17	0,96
Excluded	Severe UC (mild baseline)	Bacteria	0,68	0,71	0,66	0,17	0,97
Included	Severe UC (mild baseline)	Bacteria	0,66	0,67	0,66	0,16	0,96
Excluded	Severe UC (mild baseline)	Bacteria and biochemical	0,68	0,70	0,68	0,18	0,97
Included	Severe UC (mild baseline)	Bacteria and biochemical	0,66	0,69	0,66	0,17	0,97
Excluded	Severe UC (mild baseline)	Biochemical	0,60	0,61	0,67	0,15	0,96
Included	Severe UC (mild baseline)	Biochemical	0,57	0,57	0,67	0,15	0,95
Excluded	Severe UC (mild baseline)	Clinical	0,68	0,80	0,58	0,14	0,97
Included	Severe UC (mild baseline)	Clinical	0,64	0,75	0,57	0,14	0,97
Excluded	Severe UC (no proctitis)	All	0,71	0,73	0,68	0,28	0,95
Included	Severe UC (no proctitis)	All	0,71	0,68	0,72	0,31	0,94
Excluded	Severe UC (no proctitis)	Bacteria	0,71	0,73	0,69	0,29	0,95
Included	Severe UC (no proctitis)	Bacteria	0,70	0,67	0,72	0,31	0,94
Excluded	Severe UC (no proctitis)	Bacteria and biochemical	0,71	0,73	0,68	0,28	0,95
Included	Severe UC (no proctitis)	Bacteria and biochemical	0,71	0,67	0,73	0,32	0,94
Excluded	Severe UC (no proctitis)	Biochemical	0,58	0,56	0,69	0,25	0,92
Included	Severe UC (no proctitis)	Biochemical	0,61	0,66	0,62	0,23	0,93
Excluded	Severe UC (no proctitis)	Clinical	0,57	0,67	0,56	0,20	0,92
Included	Severe UC (no proctitis)	Clinical	0,56	0,55	0,65	0,23	0,91
Excluded	CD vs UC	Bacteria	0,73	0,77	0,61	0,49	0,86
Included	CD vs UC	Bacteria	0,73	0,77	0,61	0,50	0,85
Excluded	CD vs UC	Bacteria and biochemical	0,74	0,78	0,61	0,49	0,86
Included	CD vs UC	Bacteria and biochemical	0,75	0,76	0,64	0,52	0,85
Excluded	CD vs UC	Biochemical	0,60	0,69	0,51	0,40	0,79
Included	CD vs UC	Biochemical	0,63	0,67	0,56	0,43	0,78
Excluded	Colonic CD vs E2/3 UC	Bacteria	0,65	0,77	0,55	0,22	0,94
Included	Colonic CD vs E2/3 UC	Bacteria	0,67	0,73	0,61	0,25	0,93
Excluded	Colonic CD vs E2/3 UC	Bacteria and biochemical	0,65	0,74	0,58	0,23	0,94
Included	Colonic CD vs E2/3 UC	Bacteria and biochemical	0,66	0,73	0,60	0,25	0,93
Excluded	Colonic CD vs E2/3 UC	Biochemical	0,60	0,70	0,56	0,22	0,93
Included	Colonic CD vs E2/3 UC	Biochemical	0,63	0,73	0,54	0,23	0,93
