# Supplementary material for: Fecal Microbiome Reflects Disease State and Prognosis in Inflammatory Bowel Disease in an Adult Population-Based Inception Cohort
Source: Inflamm Bowel Dis. 2025 Apr 25;31(8):2066–80. doi: 10.1093/ibd/izaf060 (PMC12491950; doi:10.1093/ibd/izaf060)
Supplement: izaf060_suppl_Supplementary_Materials [file izaf060_suppl_supplementary_materials.docx]

**WHAT YOU NEED TO KNOW**

BACKGROUND AND CONTEXT

Gut microbiome has established associations with inflammatory bowel disease (IBD), but diagnostic and prognostic potential remains unclear in part due to previous shortcomings in study design and methodology.

NEW FINDINGS

The microbiome is linked to the current state of inflammation in IBD and risk of future severe disease. Diagnostic associations were confirmed with a global validation cohort.

LIMITATIONS

The study is limited by its sequencing method (16S), which cannot identify bacteria at species-level. Additionally, fecal samples were for some participants obtained after treatment was commenced.

CLINICAL RESEARCH RELEVANCE

Our study indicates clinical potential of the microbiome as both a diagnostic and prognostic tool, particularly with regard to early identification of patients with ulcerative colitis at high risk of developing a severe disease course. Further, we show a lack of certain health-associated bacteria in these participants, making them an interesting target in clinical trials focusing on fecal microbiota transplantation.

BASIC RESEARCH RELEVANCE

The unique microbial signatures of Crohn’s disease, ulcerative colitis, and those participants with the latter diagnosis heading for a severe disease course, suggests a significant role of the microbiome in disease development, warranting further research to understand the nature these links and unveiling underlying mechanisms.

**LAY SUMMARY**

We show that the gut bacterial flora can predict disease severity and -type better than current methods, which could in turn improve patient care for people with IBD.
